# Identification of a Divergent Environmental DNA Sequence Clade Using the Phylogeny of Gregarine Parasites (Apicomplexa) from Crustacean Hosts

**DOI:** 10.1371/journal.pone.0018163

**Published:** 2011-03-31

**Authors:** Sonja Rueckert, Timur G. Simdyanov, Vladimir V. Aleoshin, Brian S. Leander

**Affiliations:** 1 Program in Integrated Microbial Biodiversity, Departments of Botany and Zoology, Canadian Institute for Advanced Research, University of British Columbia, Vancouver, British Columbia, Canada; 2 Shimoda Marine Research Center, University of Tsukuba, Shimoda, Shizuoka, Japan; 3 Department of Invertebrate Zoology, Biological Faculty, Moscow State University, Moscow, Russian Federation; 4 Department of Evolutionary Biochemistry, Belozersky Institute for Physico-Chemical Biology, Moscow State University, Moscow, Russian Federation; Université Paris Sud, France

## Abstract

**Background:**

Environmental SSU rDNA surveys have significantly improved our understanding of microeukaryotic diversity. Many of the sequences acquired using this approach are closely related to lineages previously characterized at both morphological and molecular levels, making interpretation of these data relatively straightforward. Some sequences, by contrast, appear to be phylogenetic orphans and are sometimes inferred to represent “novel lineages” of unknown cellular identity. Consequently, interpretation of environmental DNA surveys of cellular diversity rely on an adequately comprehensive database of DNA sequences derived from identified species. Several major taxa of microeukaryotes, however, are still very poorly represented in these databases, and this is especially true for diverse groups of single-celled parasites, such as gregarine apicomplexans.

**Methodology/Principal Findings:**

This study attempts to address this paucity of DNA sequence data by characterizing four different gregarine species, isolated from the intestines of crustaceans, at both morphological and molecular levels: *Thiriotia pugettiae* sp. n. from the graceful kelp crab (*Pugettia gracilis*), *Cephaloidophora* cf. *communis* from two different species of barnacles (*Balanus glandula* and *B. balanus*), *Heliospora* cf. *longissima* from two different species of freshwater amphipods (*Eulimnogammarus verrucosus* and *E. vittatus*), and *Heliospora caprellae* comb. n. from a skeleton shrimp (*Caprella alaskana*). SSU rDNA sequences were acquired from isolates of these gregarine species and added to a global apicomplexan alignment containing all major groups of gregarines characterized so far. Molecular phylogenetic analyses of these data demonstrated that all of the gregarines collected from crustacean hosts formed a very strongly supported clade with 48 previously unidentified environmental DNA sequences.

**Conclusions/Significance:**

This expanded molecular phylogenetic context enabled us to establish a major clade of intestinal gregarine parasites and infer the cellular identities of several previously unidentified environmental SSU rDNA sequences, including several sequences that have formerly been discussed broadly in the literature as a suspected “novel” lineage of eukaryotes.

## Introduction

Environmental DNA surveys have been used to study the diversity of eukaryotic micro-organisms in different habitats (e.g., [1]–[5]). Although this method undeniably contributes greatly to our understanding of microbial eukaryotic diversity, it comes with some difficulties and uncertainties. One of these difficulties is the woefully incomplete molecular sampling of previously described eukaryotes [1]. Therefore, inferred discoveries of novel eukaryotic groups based on molecular phylotypes alone is tenuous because one must be sure that these molecular phylotypes do not belong to already described, but unsequenced, species of eukaryotes [1]. For instance, two major SSU rDNA clades of alveolates, namely Marine Alveolate Clade I and II, were demonstrated during an environmental DNA survey in the Antarctic polar front [4]. Subsequent to this study, several parasitic relatives of dinoflagellates, such as *Syndinium*, *Hematodinium* and *Amoebophrya*, were characterized at the molecular level and found to belong to Marine Alveolate Clade II; *Dubosquella*, *Ichthyodinium* and the so called ‘RP parasites’ were found to belong to Marine Alveolate Clade I [5]–[9]. Other environmental (and highly divergent) SSU rDNA sequences have been suspected to be early branching eukaryotic lineages that have yet to be identified or characterized at the cellular level [2], [4], [10]–[14]. For example, Richards & Bass [15] suggested that the highly divergent ‘DH148-5-EKD18’ (first published by Lopéz-García et al. [4]) is an uncharacterized lineage affiliated with parabasalids. Cavalier-Smith [16] was able to identify relatives (including gregarines) for some of these ‘anaerobic mystery clades’ published by Dawson & Pace [10] and Stoeck & Epstein [14]. Even though Cavalier-Smith [16] was certain that none of the sequences represent novel kingdoms or phyla the identification as gregarines for some of them was questionable due to very long branches (e.g. BOLA48). Berney et al. [1] were also able to identify relatives for supposedly novel high-level eukaryotic lineages, but for 10 sequences (including DH148-5-EKD18 and BOLA48) the possibility of representing novel high-level diversity remained. The molecular data generated in our study from gregarine apicomplexans enabled us to revisit several of these inferences with much more confidence.

Accordingly, improved molecular characterization of gregarine apicomplexans is expected to provide significant insights into the cellular identities of environmental SSU rDNA sequences. Gregarines are obligate unicellular parasites that infect the intestines, reproductive organs and body cavities of invertebrates living in terrestrial, freshwater, and marine habitats [17]. These parasites form resistant cysts that appear to be ubiquitously distributed throughout marine, freshwater and terrestrial sediments. The systematics of the group is organized around three traditional categories based more on convenience than phylogenetic relationships: eugregarines, archigregarines, and neogregarines [18]–[19]. The Eugregarinorida, in particular, is ill-defined by the absence of an asexual proliferation phase called ‘mergony’, and the species therein tend to possess trophozoites (feeding stages) with gliding motility and many longitudinal epicytic folds (e.g., *Lecudina* and *Gregarina*); however, many exceptionally divergent morphologies also exists within eugregarines (e.g., some species of *Lankesteria* and *Pterospora*). Nonetheless, this group alone contains approximately 1,650 described species and many more are thought to exist [20]–[21]. Although there is only a small proportion of described species relative to the actual number of species available to study, a vastly smaller fraction of these has been studied at the molecular level and made accessible via GenBank. Moreover, several environmental sequences have been attributed to gregarine apicomplexans, but it is unclear how many of these sequences represent gregarine species previously described with morphology alone.

Species of eugregarines have been separated into two subcategories, namely the ‘Septatorina’ and the ‘Aseptatorina’, based on the presence or absence of a visible transverse groove or ‘septum’ that divides trophozoites into a protomerite compartment and a deutomerite compartment [22]. Septate eugregarines are found in arthropods, most commonly within the intestines of insects, and over half of the available gregarine SSU rDNA sequences characterized so far (27 in total) were published last year from eugregarines isolated from insects [23]. However, the phylogenetic relationships of these eugregarine sequences have not been analyzed within the context of apicomplexans as a whole, and gregarines that inhabit the intestines of other major groups of arthropods, particularly crustaceans, have yet to be characterized at the molecular level. Currently, there are 146 described species within 15 genera of eugregarines that infect crustacean hosts, ranging from copepods to malacostracans; the majority of these gregarine species (i.e, 133) are considered septate and the minority of these species (i.e., 13) are considered aseptate [20].

In this study, we addressed the molecular phylogenetic positions of septate and aseptate eugregarines from crustacean hosts using SSU rDNA sequences, light microscopy (LM), and scanning electron microscopy (SEM): (1) one sequence from *Thiriotia pugettiae* sp. n. from the decapod *Pugettia gracilis*; (2) two sequences from *Cephaloidophora* cf. *communis*, one each from the barnacles *Balanus balanus* and *Balanus glandula*; (3) two sequences from *Heliospora* cf. *longissima*, one each from the freshwater gammarid amphipods *Eulimnogammarus verrucosus* and *Eulimnogammarus vittatus*; (4) one sequence from *Heliospora caprellae* comb. n. from the amphipod *Caprella alaskana* and (5) one sequence from *Ganymedes themistos* [24] from the amphipod *Themisto libellula*. Molecular phylogenetic analyses of these new data enabled us to re-evaluate the relationships between gregarine species isolated from arthropod hosts and to identify a diverse collection of 48 previously unidentified environmental sequences that form a robust clade consisting of all known gregarine sequences acquired from gregarines infecting crustacean hosts.

## Materials and Methods

### Collection and isolation of organisms

Four gregarine species were collected from different crustacean hosts: three from marine environments and one from a freshwater environment. *Cephaloidophora* cf. *communis* was collected from the intestines of two different barnacles: (1) *Balanus glandula* Darwin, 1854 (Cirripedia) collected from Ellis Islet (48° 51′ 48″N, 125° 06′ 23″W), Vancouver Island, Canada in June 2007 and (2) *Balanus balanus* Linnaeus, 1758 (Cirripedia) collected from the White Sea Biological Station of Moscow State University, Velikaya Salma Straight in Kandalaksha Gulf of White Sea (66°33′12″N, 33°06′17″E), Russia in 2006. *Heliospora caprellae* comb. n. was isolated from the intestines of the skeleton shrimp *Caprella alaskana* Mayer, 1903 (Amphipoda) collected from Bamfield Inlet (48° 48′ 59″N, 125° 09′ 19″W), Vancouver Island, Canada in June 2008. A dredge haul was conducted at 20 m depth near Wizard Islet (48°51′06″N, 125°09′04″W), Vancouver Island, Canada in 2009. The graceful kelp crab *Pugettia gracilis* Dana, 1851 (Brachyura) was collected from these samples and specimens of *Thiriotia pugettiae* sp. n. were isolated from the intestines of *P. gracilis*. In addition, one gregarine species *Heliospora* cf. *longissima* was isolated from the intestines of two different freshwater amphipods endemic for Lake Baikal, namely *Eulimnogammarus verrucosus* (Gerstfeldt, 1858) and *Eulimnogammarus vittatus* (Dybowski, 1874), both species collected from the settlement area Bolshiye Koty (51°54′12″N, 105°04′30″E), Lake Baikal, Russia in 2005.

The trophozoites of each species were released in seawater or physiological NaCl solution (150 mM) by teasing apart the intestines of the respective host with fine-tipped forceps under a dissecting microscope (Leica MZ6; MBS-1, LOMO). The gut material was examined under an inverted microscope (Zeiss Axiovert 200) or a stereomicoscope (MBS-1, LOMO) and parasites were isolated by micromanipulation and washed three times in either filtered seawater or physiological NaCl solution (150 mM) depending on the host species (marine/freshwater), before being examined under the light microscope or prepared for DNA extraction.

### Light and scanning electron microscopy

Differential interference contrast (DIC) light micrographs of *Cephaloidophora* cf. *communis* from *Balanus glandula* were produced by securing parasites under a cover slip with Vaseline and viewing them with an imaging microscope (Zeiss Axioplan 2) connected to a colour digital camera (Leica DC500). Light micrographs of *Heliospora caprellae* comb. n. and *Thiriotia pugettiae* sp. n. were produced using an inverted microscope (Zeiss Axiovert 200) connected to a PixeLink Megapixel color digital camera. Light micrographs of *Heliospora* cf. *longissima* were produced using a microscope (Karl Zeiss, Jena) and a Nikon Coolpix 7900 camera.

Up to 30 specimens each of the four gregarine species were prepared for scanning electron microscopy (SEM). Individuals of *C.* cf. *communis*, *H. caprellae* comb. n. and *T. pugettiae* sp. n., were deposited directly into the threaded hole of separate Swinnex filter holders, containing a 5 µm polycarbonate membrane filter (Millipore Corp., Billerica, MA), that was submerged in 10 ml of seawater within a small canister (2 cm diam. and 3.5 cm tall). A piece of Whatman No. 1 filter paper was mounted on the inside base of a beaker (4 cm diam. and 5 cm tall) that was slightly larger than the canister. The Whatman filter paper was saturated with 4% (w/v) OsO_4_ and the beaker was turned over the canister. The parasites were fixed by OsO_4_ vapours for 30 min. Ten drops of 4% (w/v) OsO_4_ were added directly to the seawater and the parasites were fixed for an additional 30 min. A 10-ml syringe filled with distilled water was screwed to the Swinnex filter holder and the entire apparatus was removed from the canister containing seawater and fixative. The parasites were washed with water, dehydrated with a graded series of ethyl alcohol, and critical point dried with CO_2_. Filters were mounted on stubs, sputter coated with 5 nm gold-palladium, and viewed under a scanning electron microscope (Hitachi S4700). Trophozoites of *C*. cf. *communis* from *Balanus balanus* and *H.* cf. *longissima* from both hosts were fixed with 2.5% (v/v) glutaraldehyde in 0.05 M cacodylate buffer (pH = 7.4) containing 1.28% (w/v) NaCl (in an ice bath, in the dark, two replacements of the fixative, each for 1 hour), rinsed three times with the same buffer, and post-fixed with 2% (w/v) OsO_4_ in the same buffer (in an ice bath for 2 hours). After dehydration in a graded series of ethyl alcohol, the gut fragments containing parasites were transferred into an ethanol/acetone mixture 1∶1 (v/v), rinsed three times in pure acetone, and critical point dried with CO_2_. The samples were mounted on stubs, sputter coated with gold-palladium, and examined with a CamScan-S2 (CamScan, UK) scanning electron microscope. Some SEM data were presented on a black background using Adobe Photoshop 6.0 (Adobe Systems, San Jose, CA).

### DNA isolation, PCR, cloning, and sequencing

Individual trophozoites of each species were isolated from the dissected hosts, washed three times in filtered seawater or in 150 mM physiological NaCl solution (for *Heliospora* cf. *longissima* from freshwater hosts), and deposited into a 1.5-ml microcentrifuge tube: 88 trophozoites of *C.* cf. *communis* from *B. glandula*, 93 trophozoites of *C.* cf. *communis* from *B. balanus*, 20 trophozoites of *H.* cf. *longissima* from *E. verrucosus*, 25 trophozoites of *H.* cf. *longissima* from *E. vittatus*, 27 trophozoites of *H. caprellae* comb. n. from *Caprella alaskana*, and 53 trophozoites of *T. pugettiae* sp. n. from *Pugettia gracilis*. DNA of *C.* cf. *communis* from *B. glandula*, *H. caprellae* comb. n. and *T. pugettiae* sp. n. was extracted using the MasterPure™ Complete DNA and RNA Purification Kit (Epicentre Biotechnologies, Madison, WI). Small subunit rDNA sequences were PCR-amplified using a total volume of 25 µl containing 1–2 µl of primer, 1–5 µl of DNA template, 18–23 µl of water and puReTaq Ready-to-go PCR beads (GE Healthcare, Quebec, Canada).

The SSU rDNA sequences from these species were amplified in one fragment using universal eukaryotic PCR primers F1 5′-GCGCTACCTGGTTGATCCTGCC-3′ and R1 5′-GATCCTTCTGCAGGTTCACCTAC-3′ [25] and internal primers designed to match existing eukaryotic SSU sequences F2 5′-AAGTCTGGTGCCAGCAGCC-3′ and R2 5′-TTTAAGTTTCAGCCTTGCG-3′. PCR was performed using MJ Mini™ Gradient Thermal Cycler (Bio-Rad) and the following protocol: After 4 cycles of initial denaturation at 94°C for 4.5 min, 45°C for 1 min and 72°C for 1.75 min, 34 cycles of 94°C for 30 sec (denaturation), 50°C for 1 min (annealing), 72°C for 1.75 min (extension), followed by a final extension period at 72°C for 10 min. PCR products corresponding to the expected size were gel isolated using the UltraClean™ 15 DNA Purification kit (MO Bio, Carlsbad, California) and cloned into the pCR 2.1 vector using the TOPO TA cloning kit (Invitrogen, Frederick, MD). Eight cloned plasmids were digested with *Eco*RI and screened for size. One or two clones for each species were sequenced with ABI big dye reaction mix using vector primers and internal primers oriented in both directions. One SSU rDNA clone was sequenced from *C.* cf. *communis* (from *B. glandula*) and from *T. pugettiae* sp. n. Two SSU rDNA clones were sequenced from *H. caprellae* comb. n., which differed by 9/1645 base pairs.

Samples of *C.* cf. *communis* from *B. balanus*, and of *H.* cf. *longissima* from *E. verrucosus* and from *E. vittatus* were treated by an alcaline lysis procedure (compare [26]). The lysates were directly used in the PCR reactions. The SSU rDNA sequences were amplified using universal eukaryotic PCR primers A 5′-GTATCTGGTTGATCCTGCCAGT-3′and B 5′-GAATGATCCWTCMGCAGGTTCACCTAC-3′ [27] in a PCR mix with a total volume of 25 µl (2 µl of primer, 1 µl of DNA template, 12 µl of water, and 10 µl of PCR solution). PCR was performed with “GenePak DNA PCR Core kit” (Isogene, Russia) using DNA Engine Dyad thermocycler (Bio-Rad) and the following protocol: initial denaturation at 95°C for 3 min, 42 cycles of 92°C for 30 sec (denaturation), 47°C for 30 sec (annealing), 72°C for 1.5 min (extension), followed by a final extension period at 72°C for 10 min. PCR products corresponding to the expected size were gel isolated using the Cytokine DNA isolation kit (Cytokine, Russia). Fragments were cloned using InsTAclone PCR Cloning Kit (Fermentas, Lithuania). One or two clones for each species were sequenced using ABI PRISM® BigDye™ Terminator v. 3.1 reagent kit followed by an analysis of the reaction products with a DNA Genetic Analyzer (ABI PRISM 3730, Applied Biosystems). One SSU rDNA clone was sequenced from *C.* cf. *communis* (from *B. balanus*) and from *H.* cf. *longissima* (from *E. verrucosus*). Two SSU rDNA clones were sequenced from *H.* cf. *longissima* (from *E. vittatus*), which differed by 3/886 base pairs.

The new SSU rDNA sequences were initially identified by BLAST analysis and subsequently verified with molecular phylogenetic analyses (GenBank Accession numbers: *Thiriotia pugettiae* sp. n. HQ876006, *Cephaloidophora* cf. *communis* from *Balanus balanus* HQ891113, *Cephaloidophora* cf. *communis* from *Balanus glandula* HQ876008, *Heliospora* cf. *longissima* from *Eulimnogammarus verrucosus* HQ891114, *Heliospora* cf. *longissima* from *Eulimnogammarus vittatus* HQ891115, *Heliospora caprellae* comb.n. HQ876007).

### Molecular phylogenetic analysis

The six new SSU rDNA sequences (*C.* cf. *communis* from *B. glandula*, *C.* cf. *communis* from *B. balanus*, *H. caprellae* comb. n., *T. pugettiae* sp. n., *H.* cf. *longissima* from *E. verrucosus*, and *H.* cf. *longissima* from *E. vittatus*) and the sequence of *Ganymedes themistos* from *Themisto libellula* (planktonic hyperiid amphipod) were aligned with 76 other SSU rDNA sequences, representing the major lineages of apicomplexans, using MacClade 4 [28] and visual fine-tuning; gaps and ambiguously aligned bases were excluded from the 82-taxon alignment resulting in 1,006 unambiguously aligned sites.A NEXUS file of this alignment is available upon request.

PhyML [29]–[30] was used to analyze the dataset (one heuristic search) with maximum-likelihood (ML) using a general-time reversible (GTR) model of base substitutions [31] that incorporated invariable sites and a discrete gamma distribution with eight rate categories (GTR + I + Gamma model). The GTR model was selected using the program MrAIC 1.4.3 with PhyML (http://www.abc.se/~nylander/mraic/mraic.html), and model parameters were estimated from the original dataset (α  =  0.514, proportion of invariable sites  =  0.099). ML bootstrap analyses were conducted with the same settings described above (100 pseudoreplicates; one heuristic search per pseudoreplicate).

We also examined the 82-taxon data set twice with Bayesian analysis using the program MrBayes 3.0 [32]–[33]. The program was set to operate with GTR, a gamma-distribution, and four Monte Carlo Markov chains starting from a random tree (MCMC; default temperature  =  0.2). A total of 2,000,000 generations were calculated with trees sampled every 50 generations and with a prior burn-in of 200,000 generations (2,000 sampled trees were discarded; burn-in was checked manually). A majority-rule consensus tree was constructed from 38,001 post-burn-in trees. Posterior probabilities correspond to the frequency at which a given node is found in the post-burn-in trees.

The seven gregarine SSU rDNA sequences from crustacean hosts were also aligned with 48 environmental SSU rDNA sequences forming a 55-taxon alignment. The environmental sequences were selected from NCBI nucleotide database (GenBank) using BLAST search. Gaps and ambiguously aligned bases were excluded from the 55-taxon alignment resulting in 1,486 unambiguously aligned sites. The alignment was examined with maximum-likelihood (ML) using a general-time reversible (GTR) model of base substitutions that incorporated invariable sites and a discrete gamma distribution with eight rate categories (GTR + I + Gamma model). PhyML was used to analyse the dataset and model parameters were estimated from the original dataset. Nonparametric bootstrap analyses were performed with 100 replications. The 55-taxon dataset was also examined with Bayesian analysis using the program MrBayes 3.0. The program was set to operate using the following parameters: nst = 6, ngammacat = 8, rates = invgamma, covarion = yes; parameters of Monte Carlo Marcov chains: nchains = 4, nruns = 4, ngen = 3000000, samplefreq = 1000, burnin = 2000; average standard deviation of split frequencies  =  0.02 is reached in the end of the calculations.

### Nomenclatural Acts

The electronic version of this document does not represent a published work according to the International Code of Zoological Nomenclature (ICZN), and hence the nomenclatural acts contained in the electronic version are not available under that Code from the electronic edition. Therefore, a separate edition of this document was produced by a method that assures numerous identical and durable copies, and those copies were simultaneously obtainable (from the publication date noted on the first page of this article) for the purpose of providing a public and permanent scientific record, in accordance with Article 8.1 of the Code. The separate print-only edition is available on request from PLoS by sending a request to PLoS ONE, Public Library of Science, 1160 Battery Street, Suite 100, San Francisco, CA 94111, USA along with a check for $10 (to cover printing and postage) payable to “Public Library of Science”.

The online version of the article is archived and available from the following digital repositories: PubMedCentral (www.pubmedcentral.nih.gov/), and LOCKSS (http://www.lockss.org/lockss/). In addition, this published work and the nomenclatural acts it contains have been registered in ZooBank, the proposed online registration system for the ICZN. The ZooBank LSIDs (Life Science Identifiers) can be resolved and the associated information viewed through any standard web browser by appending the LSID to the prefix “http://zoobank.org/”.

The LSID for this publication is: (urn:lsid:zoobank.org:pub:AC620E06-6CD4-4846-A3DC-8DEABB5CB461).

## Results

### Morphological observations

#### *Cephaloidophora* cf. *communis*

**Figure 1:**
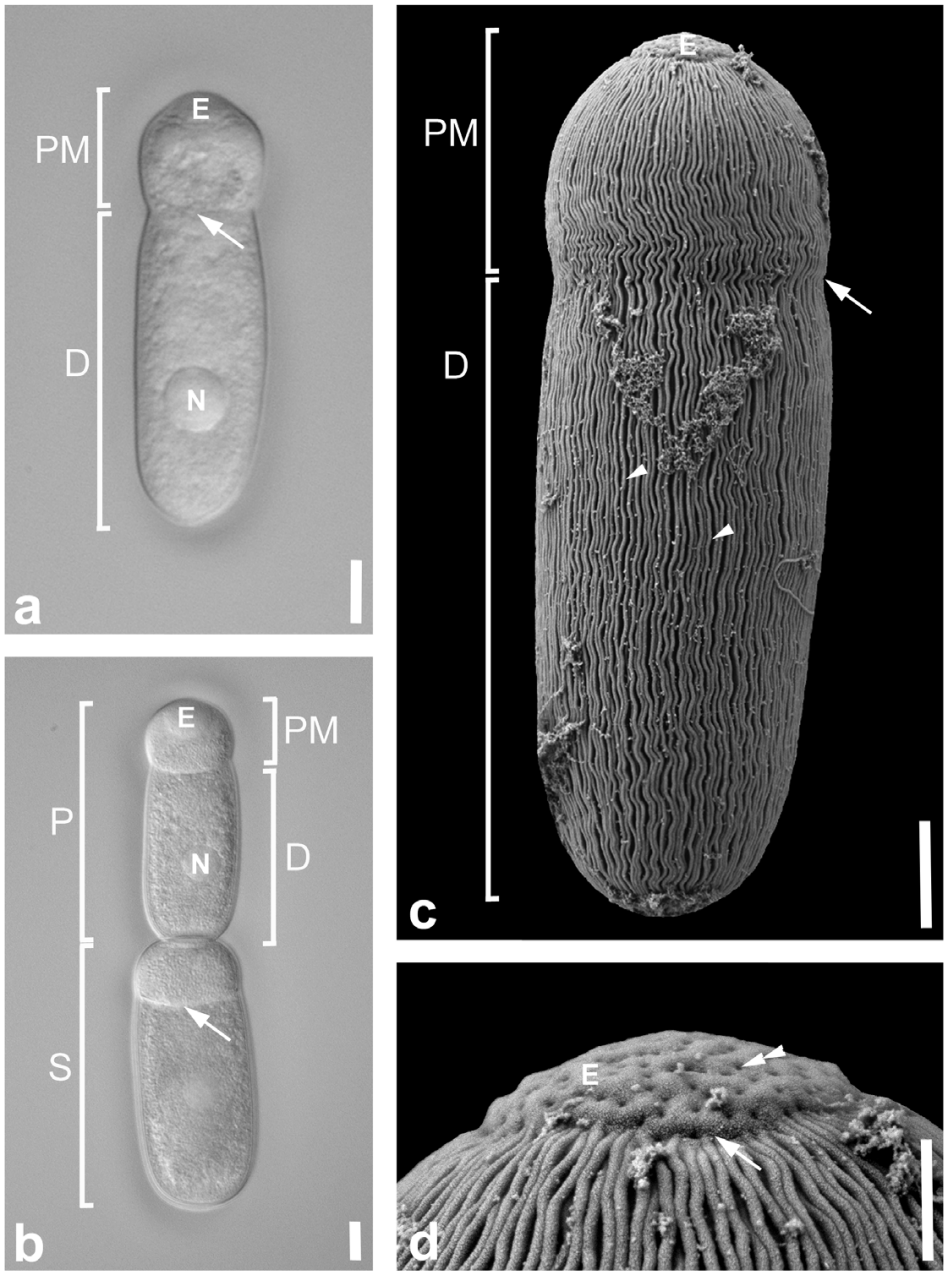
Differential interference contrast (DIC) light micrographs and scanning electron micrographs (SEM) showing the general morphology and surface ultrastructure of the gregarine *Cephaloidophora* cf. *communis*. **a.** Trophozoite showing the cell organization of the septate gregarine. The cell is divided by a septum (arrow) into the protomerite (PM) with the epimerite (E) at the anterior end and the deutomerite (D) with the spherical nucleus (N). **b.** An association of two gregarines paired up in caudo-frontal syzygy. The anterior trophozoite is the primite (P), while the posterior trophozoite is the satellite (S). **c.** SEM showing a trophozoite with epimerite (E), protomerite (PM) and deutomerite (D). Except for the mucron the trophozoite is covered with epicytic folds (arrowheads). There is an indentation (arrowhead) visible at the level of the septum, but the epicytic folds are continuous throughout the trophozoite. **d.** Higher magnification SEM of the anterior end of a trophozoite with the epimerite (E) free of epicytic folds. There is a visible junction between the protomerite with folds and the epimerite without folds (arrow). Surface pores (double arrowhead) are evenly distributed across the epimerite. Scale bars: Figs. 1a–b, 10 µm; Fig. 1c, 3 µm; Fig. 1d, 1 µm.

Trophozoites were isolated from two different barnacle species, *B. balanus* and *B. glandula*, and conformed in morphology to the original description of *C. communis* from several different species of *Balanus*, namely *B. amphitrite*, *B. eburneus*, *B. crenatus*, *B. glandula*, *B. cariosus*, and *B. improvisus* [34]–[35]. The cells were cylindrical in shape with the typical septate eugregarine morphology consisting of a protomerite and deutomerite (Figure 1a). Trophozoite were 46.7 µm (24.3–80.0 µm, n  =  24) long and 17.8 µm (8.3–33 µ µm, n  =  24) wide. The epimerite was rudimental and rounded (diameter  =  7.6 µm, n  =  15) and contained an even distribution of surface pores with no distinct pattern when viewed under the SEM (Figures 1c–d). The protomerite was 11.3 µm (5.2–20.0 µm, n  =  23) long and 15.4 µm (7.8–26.0 µm, n  =  23) wide; the deutomerite was longer than the protomerite with a length of 34.0 µm (17.8–67.0 µm, n  =  23) and a width of 17.8 µm (8.3–33 µm, n  =  24). The septum was clearly visible under the LM (Figures 1a–b) and less robust under the SEM. The posterior end of the deutomerite was rounded. The spherical nucleus [10 µm (9–10 µm) in diameter, n = 10] was situated in the middle of the deutomerite or sometimes shifted toward either the posterior or anterior end. All trophozoites were brownish in colour under the LM, reflecting an accumulation of amylopectin granules within the cytoplasm. Mature trophozoites (or gamonts) pair up in caudo-frontal syzygy in which the anterior gregarine is called the primite and the posterior gregarine is called the satellite (Figure 1b). Two individuals in syzygy were 106.4 µm (55.0–142.0 µm, n  =  7) long. There was no obvious pattern of the satellite being conspicuously smaller or bigger than the primite. SEM micrographs demonstrated that the protomerite and the deutomerite were continuously covered in epicytic folds with a slight indentation at the level of the septum. The density of folds was up to 6 folds/micron (Figure 1c). Single trophozoites and two individuals in syzygy were capable of gliding movements.

#### *Heliospora caprellae* comb. n

**Figure 2:**
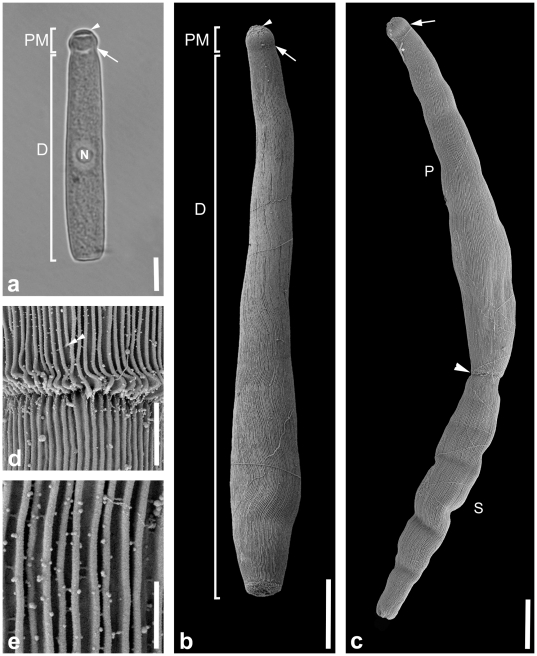
Differential interference contrast (DIC) light micrograph and scanning electron micrographs (SEM) showing the general morphology and surface ultrastructure of the gregarine *Heliospora caprellae* comb. n. **a.** DIC micrograph showing the epimerite (arrowhead), small and rounded protomerite (PM) and the elongated deutomerite (D). The arrow marks the septum between the protomerite and deutomerite. The nucleus (N) is located in the middle of the deutomerite. **b.** SEM of a single trophozoite with a small protomerite (PM) and a long deutomerite (D) that is wider at the posterior end than at the anterior end. The deutomerite ends in a blunt posterior tip. A slight indentation (arrow) is visible between the protomerite and deutomerite in the area of the septum. Epicytic folds cover the whole trophozoite except for the epimerite (arrowhead) and show an undulating pattern. **c.** SEM of an association consisting of two trophozoites (or gamonts). The anterior primite (P) has a visible indentation in the area of the septum (arrow) and connects to the anterior end (arrowhead) of the posterior satellite (S). **d.** Higher magnification SEM of the junction between primite (P) and satellite (S). Some of the epicytic folds (double arrowhead) terminate before they reach the posterior end of the primite. **e.** High magnification SEM of the epicytic folds. The density of the folds is 5 folds/micron. Scale bars: Fig. 2a, 20 µm; Fig. 2b, 14 µm; Fig. 2c, 11 µm; Fig. 2d, 2 µm; Fig. 2e, 1 µm.

The trophozoites of this septate eugregarine were highly abundant within the intestines of the skeleton shrimp *Caprella alaskana* and could take up the whole volume of the intestinal lumen. The trophozoites were very active and capable of gliding. Trophozoites were cylindrical and 82.2 µm (21.7–128.0, n  =  17) long and 13.3 µm (6.0–21.0 µm, n  =  17) wide (Figures 2a–b). The epimerite was rudimental, rounded and about 6.6 µm (1.7–10.0 µm, n  =  11) in diameter (Figures 2a–b). The protomerite was short and rounded with a length of 5.5 µm (2.3–10.0 µm, n  =  17) and a width of 9.0 µm (4.8–16.0 µm, n  =  17). The septum was clearly visible under the LM (Figure 2a) and less conspicuous under the SEM (Figures 2b–c). The deutomerite was long [75.5 µm (18.9–115.0 µm), n  =  17], and its width was either constant or became wider near the posterior end [13.3 µm (6.0–21.0 µm, measured at the widest part), n  =  17] (Figures 2a–c). The posterior end of the deutomerite was blunt (Figures 2a–b). The nucleus measured 15.8×13.7 µm (15.0–19.0×12.0–15.0 µm, n  =  6) and was usually situated in the middle of the deutomerite or slightly shifted toward the posterior end. All trophozoites were brownish in colour under the LM, reflecting an accumulation of amylopectin granules within the cytoplasm. The SEM showed that epicytic folds covered the protomerite and the deutomerite, and in some trophozoites the folds were undulating in their arrangement (Figure 2b). There was a visible junction between the primite and the satellite of associated trophozoites (or gamonts) (Figure 1d). The density of the folds was up to 5 folds/micron (Figure 2d). Single trophozoites and two individuals in syzygy were capable of gliding movements.

#### *Heliospora* cf. *longissima*

**Figure 3:**
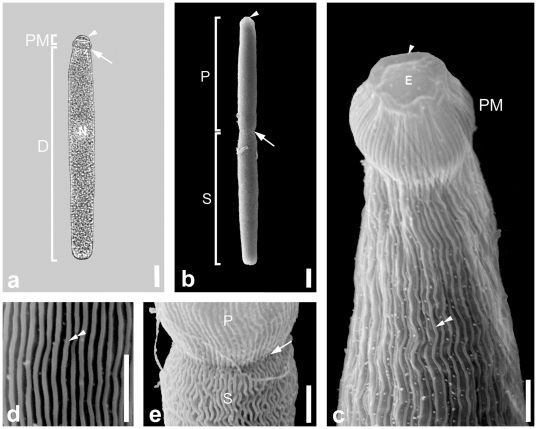
Light micrograph (LM) and scanning electron micrographs (SEM) showing the general morphology and surface ultrastructure of the gregarine *Heliospora* cf. *longissima*. **a.** LM of a trophozoite with an elongated deutomerite (D), a short protomerite (PM), and an epimerite (arrowhead). The septum (arrow) is clearly visible. The spherical nucleus (N) is situated in the middle of the deutomerite. **b.** SEM of an association of two trophozoites in caudo-frontal syzygy. The junction (arrow) between primite (P) and satellite (S) is visible. The arrowhead marks the epimerite at the anterior end of the primite. **c.** Higher magnification SEM of the anterior end of the trophozoite with a bulb-like protomerite (PM). The epimerite (arrowhead) shows a prominent collar-like margin (arrowhead). Epicytic folds (double arrowhead) cover the protomerite and deutomerite. **d.** High magnification SEM of the epicytic folds (double arrowheads). The density of folds is around 3 folds/micron. **e.** Higher magnification SEM of the area between the primite (P) and satellite (S) of an association of two trophozoites. The junction (arrow) between the two trophozoites is clearly visible. Scale bars: Fig. 3a, 20 µm; Fig. 3b, 10 µm; Figs. 3c–e, 3 µm.

Trophozoites of this septate eugregarine were isolated from the intestines of two different hosts: *Eulimnogammarus verrucosus* and *E. vittatus*. The trophozoites were long and slender and conformed in overall morphology with the original description of *Heliospora longissima* (e.g. [36]). Trophozoites were 154.0 µm (57.9–273.0 µm, n  =  16) long and 17.0 µm (9.6–25.0 µm, n  =  16) wide (Figure 3a). Mature trophozoites (or gamonts) pair up in caudo-frontal syzygy, where the primite and satellite were approximately the same size (Figure 3b). Individuals in syzygy consisting of two or three specimens were about 253.2 µm (126.4–380.0 µm, n  =  2) long. The epimerite of the trophozoites was 6.9 µm (5.2–10.0 µm, n  =  3) in diameter and showed a prominent collar-like margin under the SEM (Figure 3c). The protomerite was short and rounded, about 8.5 µm (6.0–11.0 µm, n  =  8) long and 10.4 µm (7.6–15.0, n  =  8) wide. The septum was clearly visible under the LM and the SEM (Figures 1a–c). The deutomerite was long and slender with a length of 158.8 µm (64.3–200.0 µm, n  =  7) and a width of 18.3 µm (11.9–25.0 µm, n  =  7). The posterior end of the deutomerite was blunt. The spherical nucleus (15 µm in diameter, n  =  1) was situated in the middle of the deutomerite or slightly shifted to the anterior end. All trophozoites were brownish in colour under the LM, reflecting an accumulation of amylopectin granules within the cytoplasm. The SEM showed that epicytic folds covered the protomerite and deutomerite (Figures 3d–e). The density of the folds was up to 3 folds/micron (Figure 3d). Single trophozoites and associations of two or more individuals in syzygy were capable of gliding movements.

#### *Thiriotia pugettiae* sp. n

**Figure 4:**
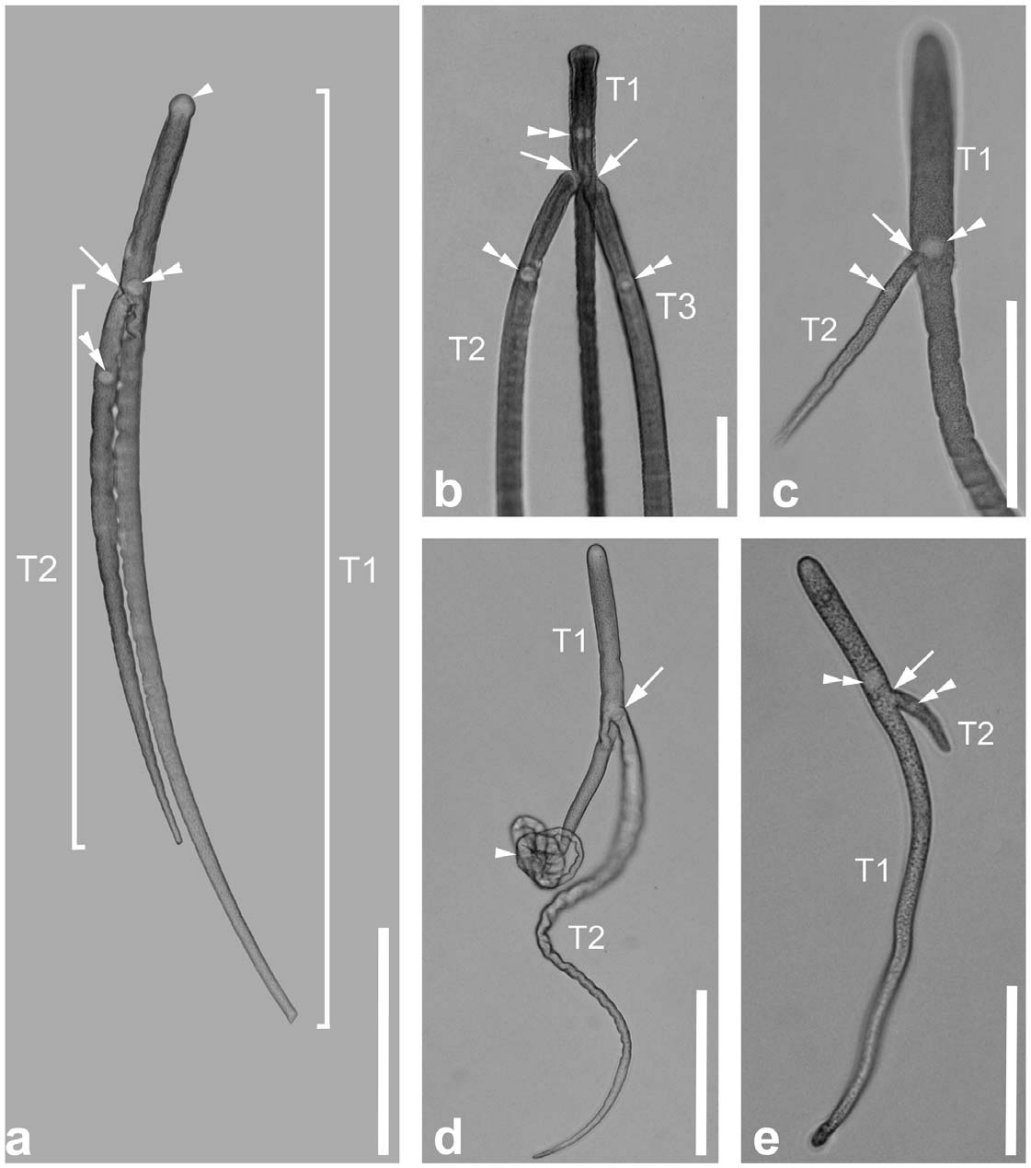
Differential interference contrast (DIC) light micrographs showing the general morphology of the gregarine *Thiriotia pugettiae* sp. n. **a.** DIC micrograph showing an association of two trophozoites. Trophozoite 1 (T1) is longer than trophozoite 2 (T2). The arrowhead marks the slightly broadened anterior tip with the mucron area free of amylopectin. The ellipsoidal nucleus (double arrowheads) is located in the anterior third of the cell in both trophozoites. The attachment site (arrow) of trophozoite 2 is at the level of the nucleus of trophozoite 1. **b.** Higher magnification view of the anterior end of an association consisting of three trophozoites. The attachment sites (arrows) of trophozoite 2 (T2) and trophozoite 3 (T3) are right behind the nucleus (double arrowhead) of trophozoite 1 (T1). **c.** Higher magnification view of an association consisting of two smaller trophozoites. The attachment site (arrow) of the much smaller trophozoite 2 (T2) is at the level of the nucleus (double arrowhead) of trophozoite 1 (T1). **d.** DIC micrograph of two trophozoites. The attachment site of trophozoite 2 (T2) is marked by an arrow. The posterior half of trophozoite 1 (T1) is curled up (arrowhead), a condition that was often recognized when trophozoites were covered in host gut material. **e.** DIC micrograph of one of the smallest documented trophozoites in an association. The attachment site (arrow) of very small trophozoite 2 (T2) is right behind the nucleus (double arrowhead) of trophozoite 1 (T1). Trophozoite 2 (T2) was only 40 µm long. Scale bars: Fig. 4a, 200 µm; Fig. 4b, 100 µm; Fig. 4c–d, 150 µm; Fig. 4e, 80 µm.

**Figure 5:**
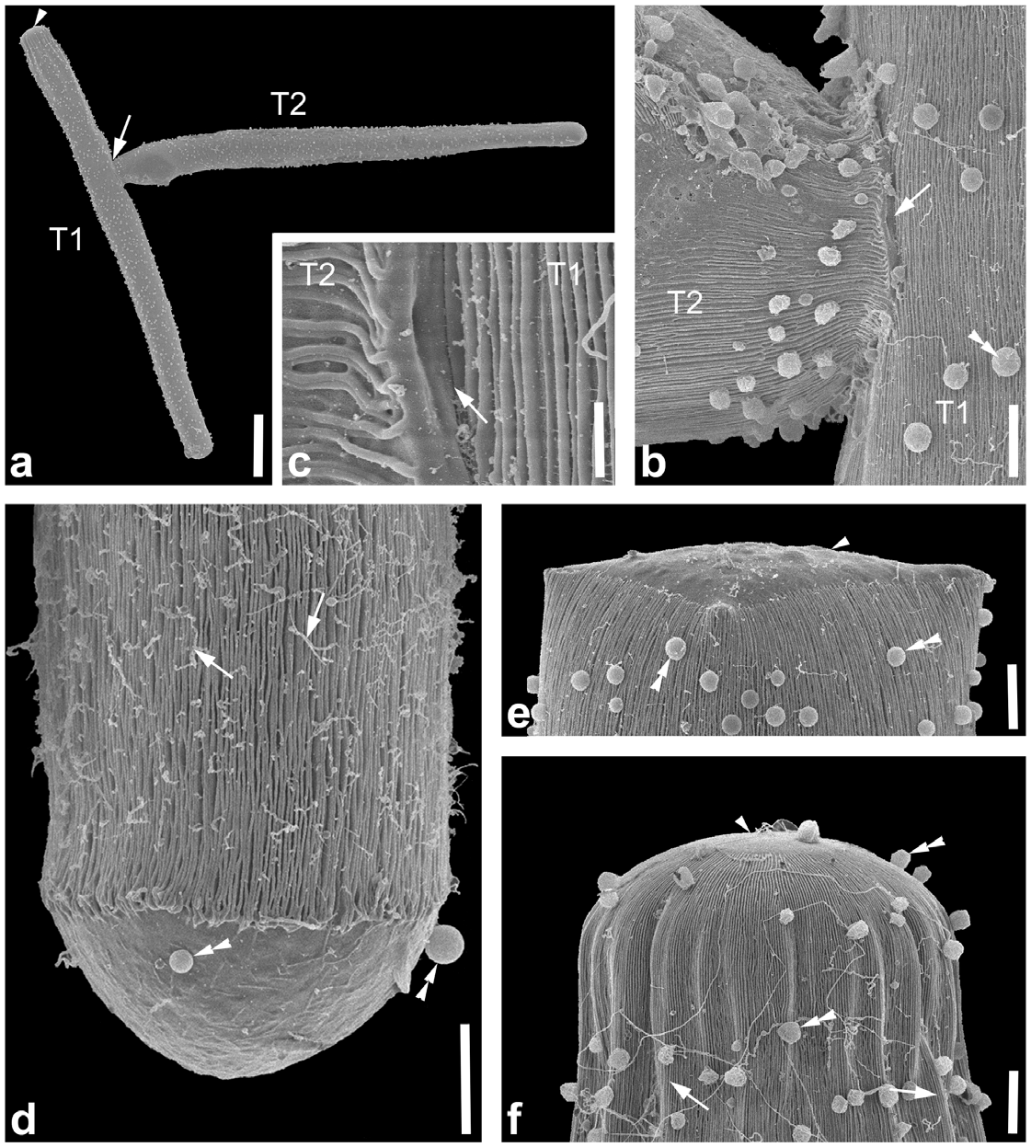
Scanning electron micrographs (SEM) showing the general morphology and surface ultrastructure of the gregarine *Thiriotia pugettiae* sp. n. **a.** SEM of an association of two trophozoites or gamonts in latero-frontal syzygy. The attachment site (arrow) of trophozoite 2 (T2) is located in the anterior half of trophozoite 1 (T1). The arrowhead marks the anterior mucron. **b.** Higher magnification SEM of the attachment site (arrow) of the two trophozoites. The double arrowhead marks knob-like structures on the trophozoite surface that is inferred to be secreted cell material associated with cell decay. **c.** High magnification SEM of the attachment site showing a smooth junction (arrow) between trophozoite 1 (T1) and trophozoite 2 (T2). **d.** SEM of the posterior end of a trophozoite. The trophozoite itself is covered in epicytic folds, but the posterior rounded tip is free of folds. Knob-like structures (double arrowheads) on the surface of the posterior tip is inferred as secreted material associated with cell decay. **e.** Higher magnification SEM of the anterior end of a trophozoite showing a mucron area (arrowhead) that is broadened and flattened due to a previous attachment to another trophozoite. The cell is also showing the knob-like structures of extruded cell material. **f.** High magnification SEM of the anterior end of a trophozoite showing the mucron (arrowhead) free of epicytic folds, while the rest of the trophozoite is entirely covered in epicytic folds. Double arrowheads mark the knob-like structures of extruded cell material. Restricted to the anterior end are some larger (super) folds (arrows) in the cell cortex. Scale bars: Fig. 5a, 60 µm; Fig. 5b, 5 µm; Fig. 5c, 1 µm; Figs. 5d–f, 5 µm.

Trophozoites of this novel aspetate species were extremely long and slender with a mean length of 1.25 mm (0.04–2.11 mm, n  =  35) and a width of 24.6 µm (8.0–37.9 µm, n  =  36) measured at the level of the nucleus (Figure 4a). The anterior end was rounded, while the trophozoite tapered gradually along its length into a pointed posterior tip (Figure 4a). Some trophozoites had a slight indentation at the anterior tip (Figures 4a–b). The spherical to ellipsoidal nucleus [17.7 (5.0–20.0) µm in diameter, n  =  31] was situated in the anterior half of the trophozoite. Mature trophozoites (or gamonts) paired up in latero-frontal syzygy (Figures 4a–e). The attachment site was either at the level of the nucleus (Figures 4a–d) or shifted slightly posterior to the nucleus (Figs 4b, e), and the syzygy stage consisted of two or three individuals. In most cases the attached trophozoite was smaller than the main trophozoite (Figures 4a, c–e). The pattern of gamont pairing during syzygy was not restricted to similar sized trophozoites; some association occurred between trophozoites (or gamonts) with very different sizes (Figures 4d, e). All trophozoites were brownish in colour under the LM reflecting an accumulation of amylopectin granules within the cytoplasm. SEM micrographs showed the presence of longitudinal epicytic folds over the entire cell surface, except the mucron and the posterior tip (Figures 5b, d–f). The density of folds was around 5 folds/micron. Knob-like exuded material on the surface of the trophozoites reflected the relative fragility of these very large cells (Figures 5a–b, e–f). The trophozoites were capable of gliding movements and were also able to curl up in little balls (Figure 4d).

### Molecular phylogenetic analyses

**Figure 6:**
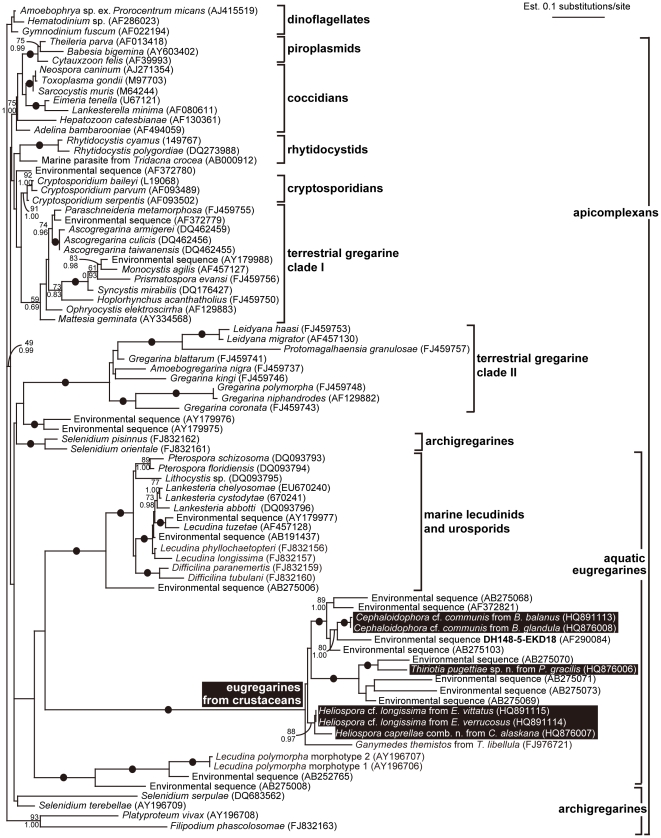
Maximum likelihood (ML) tree of apicomplexans inferred using the GTR + Γ + I model of substitution on an alignment of 82 small subunit (SSU) rDNA sequences and 1,006 unambiguously aligned sites (-ln L  =  17763.96233, α  =  0.514, proportion of invariable sites  =  0.099, eight rate categories). Numbers at the branches denote ML bootstrap percentage (top) and Bayesian posterior probabilities (bottom); values provided are higher than 65% (i.e., the absence of values reflect statistical support below 65%). Black dots on branches denote Bayesian posterior probabilities and bootstrap percentages of 95% or higher. The six sequences derived from this study are highlighted with black boxes.

Phylogenetic analyses of the 82-taxon data set, using dinoflagellates as an outgroup, resulted in a weakly supported backbone. The SSU rDNA sequences from apicomplexans clustered into six major clades: (1) a clade consisting of coccidians and piroplasmids; (2) a clade consisting of marine rhytidocystids; (3) a clade consisting of cryptosporidians; (4) a clade consisting of neogregarines and a few terrestrial eugregarines (e.g., *Monocystis* and *Paraschneideria*), forming “Terrestrial gregarine clade I”; (5) a clade consisting of several terrestrial septate eugregarines from insects, forming “Terrestrial gregarine clade II”; and (6) a clade consisting of marine and freshwater eugregarines (i.e., “aquatic eugregarines”) (Figure 6). The SSU rDNA sequences from (marine) archigregarines branched in several different positions along the unresolved backbone and, therefore, formed a polyphyletic (or paraphyletic) assemblage (Figure 6). There were several strongly supported subclades within the more inclusive “aquatic eugregarines” clade: (1) a clade consisting of marine lecudinids and urosporids (e.g., *Pterospora*, *Lankesteria*, *Difficilina*, and *Lecudina*), (2) a clade consisting of *Lecudina polymorpha* and two environmental sequences, and (3) a novel clade consisting of all eugregarines isolated from the intestines of marine and freshwater crustaceans (*Cephaloidophora*, *Heliospora*, *Thiriotia*, and *Ganymedes*) and several environmental sequences (Figure 6) for which we propose the name Cephaloidophoroidea.

**Figure 7:**
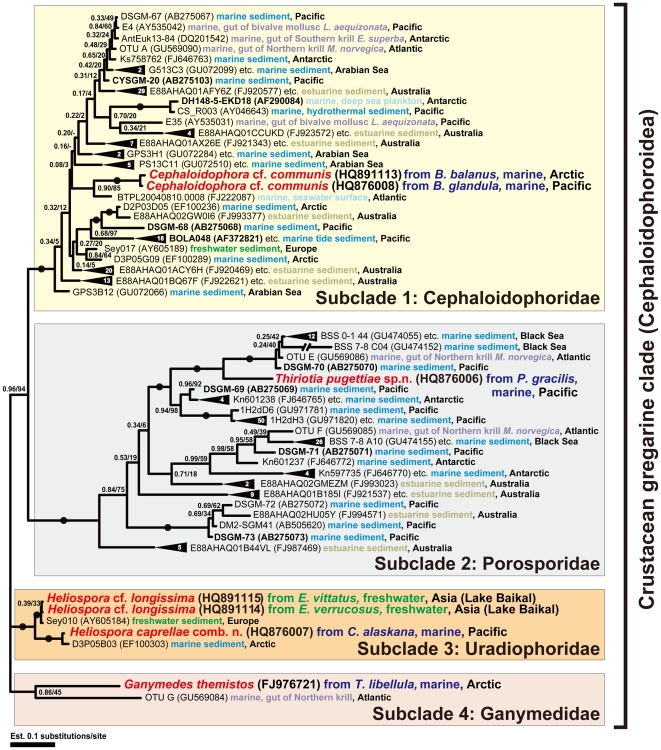
Bayesian inference (BI) tree of the studied crustacean gregarines and 48 related environmental sequences inferred using alignment of 1486 unambiguously aligned sites. Numbers at the branches denote Bayesian posterior probabilities (front) and ML bootstrap percentage (back). Black dots on branches denote Bayesian posterior probabilities and bootstrap percentages of 95% or higher. Black triangles denote groups of highly-similar sequences, numerals inside the triangles denote the number of members of such groups. The environmental sequences shown on Fig. 6 are bolded. Names of the identified gregarine species from crustacean hosts are red, samples from marine environments are blue (light-blue  =  planktonic; cyan  =  benthic), samples from the guts of marine invertebrates are purple (light-purple  =  obtained as common gut contents; dark-purple  =  obtained as identified specific gut parasites), estuarine samples are olive and samples from freshwater environments are green. The four subclades of the crustacean gregarines are color-coded according to their putative family affiliation.

Bayesian phylogenetic analysis of the 55-taxon data set that focussed on all of the gregarines isolated from crustaceans and the associated environmental sequences (Table S2) resulted in four subclades, three strongly supported clades plus one clade with only two species (Figure 7): (1) a subclade consisting of *Cephaloidophora* and related environmental sequences (2) a subclade consisting of *Thiriotia* and environmental sequences derived from marine and estuarine sediments (3) a subclade consiting of *Heliospora* spp. and environmental sequences derived from marine and freshwater sediments and (4) a subclade consisting of *Ganymedes themistos* and a sequence from the gut contents of Northern krill *Meganyctiphanes norvegica.* There are several more gregarine-related sequences in GenBank than are shown in Figure 7; however, they are identical or almost identical to at least one of the sequences included in the analyses (i.e., one representative of each cluster of near identical sequences was chosen for the analysis, see supporting information Table S1). The overwhelming majority of the environmental clones are sequences derived from marine sediments from very different habitats including the intertidal zone and deep-sea habitats (e.g. microbial mats in the methane cold seep area and hydrothermal vents). In addition, there are five sequences derived from the complete gut contents of Northern and Antarctic krill (*Meganyctiphanes norvegica* and *Euphausia superba*), two sequences from the gut contents of a marine bivalve mollusc *Lucinoma aequizonata,* two sequences from Komokiacea tests, two sequences from marine plankton samples, and two sequences from sediments taken from a river. All of these sequences represent a wide geographical distribution in various climate zones such as the Arctic, Antarctic, subtropical and moderate Pacific Ocean, subtropical Indian Ocean, temperate Atlantic Ocean, Australien estuaries, and continental fresh waters of Europe (Switzerland) and Asia (Lake Baikal).

### Comparison of SSU rDNA sequences from gregarines and associated environmental sequences

Pair-wise distance calculations using the Kimura 2-parameter model were performed on the sequences derived from *Cephaloidophora* cf. *communis* from different hosts, *Heliospora* cf. *longissima* from different hosts, *Heliospora* cf. *longissima*, and *H. caprellae* [37]. The sequences of *Cephaloidophora* cf. *communis* from *Balanus balanus* and *B. glandula* were nearly identical and only differed by 18/1604 bp (1.1% sequence divergence) and by 2 indels. The results were similar for the two sequences of *Heliospora* cf. *longissima* from *Eulimnogammarus vittatus* and *E. verrucosus*; these sequences differed by 5/1600 bp (0.3% sequence divergence). By contrast, a comparison of 1,606 bp between the SSU rDNA sequences derived from *Heliospora caprellae* (isolated from *C. alaskana*) and from *H.* cf. *longissima* (isolated from *E. verrucosus* and *E. vittatus*) resulted in 129 different bp and 16 indels. A pair-wise distance calculation of 1,590 base pairs (excluding the indels) resulted in 8.6% sequence divergence between the sequences of these two gregarine species. Similar pair-wise distance calculations were performed for four of the five gregarine sequences and four closely associated environmental sequences (*Cephaloidophora* cf. *communis* and BTPL20040810.0008, *Heliospora* cf. *longissima* and Sey010, *H. caprellae* and D3P05B03, *Thiriotia pugettiae* and DSGM-70). *Cephaloidophora* cf. *communis* (both sequences) and its closely related environmental sequence were 12.4% dissimilar; *Thiriotia pugettiae* and its closely related environmental sequence was 15.6% dissimilar. Sequence divergence between the two *H.* cf. *longissima* sequences and the environmental sequence Sey010 from freshwater sediment of a river in Switzerland was 0.41%. A sequence divergence of 1.1% was found between the sequence of *H. caprellae* and the environmental sequence D3P05B03 from intertidal sediments of Greenland.

### Formal taxonomic description

Phylum Apicomplexa Levine, 1970

Subphylum Sporozoa Leuckart, 1879

Class Gregarinea J.A.O. Bütschli, 1882, stat. nov. Grassé, 1953

Order: Eugregarinorida Léger, 1900

Superfamily Cephaloidophoroidea superfam. n., Simdyanov and Aleoshin,

**Diagnosis.** Eugregarinorida, septate and aseptate forms parasitizing the intestines of crustaceans. Syzygy early, usually caudo-frontal, rarely of another type, sometimes consisting of more than two partners. Molecular data: robust SSU rDNA clade.

Family Porosporidae Labbé, 1899

Genus *Thiriotia* Desportes, Vivarès and Théodoridès 1977

*Thiriotia pugettiae* sp. n., Rueckert and Leander

urn:lsid:zoobank.org:act:28F1D8BA-566C-469D-8E96-9531765C1381

**Diagnosis.** Trophozoites are extremely long and slender 0.04–2.11 mm long (mean length 1.25 mm) and 8.0–37.9 µm wide (mean width 24.6 µm). The anterior end is rounded, and the trophozoite tapers gradually along its length into a pointed posterior tip. The spherical to ellipsoidal nucleus (5.0–20.0 µm in diameter) is situated in the anterior half of the trophozoite. Trophozoites pair up in latero-frontal syzygy. The satellite attachment site is located at the level of the nucleus, or slightly shifted posteriorly. Syzygy is formed by two or three trophozoites (or gamonts). Trophozoites are brownish in colour under the LM due to the accumulation of amylopectin granules within the cytoplasm. Longitudinally oriented epicytic folds are present on the entire cell surface, except the mucron and the posterior tip. The trophozoites are capable of gliding movements and are able to curl up into spirals or balls.

**DNA sequence.** The SSU rDNA sequence; GenBank Accession No. HQ876006.

**Type locality.** Wizard Islet (48°51′6″N, 125°09′4″W) at a depth of 20 m, near Bamfield Marine Sciences Centre, Vancouver Island, Canada.

**Type habitat.** Marine.

**Type host.** *Pugettia gracilis* Dana, 1851 (Arthropoda, Crustacea, Malacostraca, Decapoda, Brachyura, Epialtidae).

**Location in host.** Intestinal lumen.

**Iconotype.** Figure 4a.

**Hapantotype.** Parasites on gold sputter-coated SEM stubs have been deposited in the Beaty Biodiversity Museum (Marine Invertebrate Collection) at the University of British Columbia, Vancouver, Canada.

**Etymology.** The species name *pugettiae* refers to the genus of the crustacean type host *Pugettia gracilis* Dana, 1851

Family Uradiophoridae Grassé, 1953

Genus *Heliospora* Goodrich 1949

*Heliospora caprellae* comb. n., Rueckert and Leander

**Diagnosis.** Trophozoites are cylindrical and 21.7–128.0 µ long (mean length 82.2 µm) and 6.0–21.0 µm wide (mean width 13.3 µm). The protomerite is short and rounded with a length of 2.3–10.0 µm (mean length 5.5 µm) and a width of 4.8–16.0 µm (mean width 9.0 µm), bearing the rudimental epimerite at the anterior tip. The septum is clearly visible. The deutomerite is 18.9–115.0 µm (mean length 75.5 µm) long, and its width is either constant or becomes wider near the posterior end [6.0–21.0 µm (mean width 13.3 µm, measured at the widest part). The nucleus is situated in the middle of the deutomerite or slightly shifted toward the posterior end and measures 15.8×13.7 µm (15.0–19.0×12.0–15.0). Trophozoites are brownish in colour reflecting an accumulation of amylopectin granules within the cytoplasm. Epicytic folds cover the protomerite and the deutomerite. Single trophozoites and associations of trophozoites are capable of gliding movements.

**DNA sequence.** The SSU rDNA sequence; GenBank Accession No. HQ876007.

**Type locality.** Bamfield Inlet (48° 48′ 59″N, 125° 09′ 19″W), near Bamfield Marine Sciences Centre, Vancouver Island, Canada.

**Type habitat.** Marine.

**Type host.** *Caprella alaskana* Mayer, 1903 (Arthropoda, Crustacea, Malacostraca, Amphipoda, Corophiidea, Caprellidae).

**Location in host.** Intestinal lumen.

**Iconotype.** Figure 2a.

**Hapantotype.** Parasites on gold sputter-coated SEM stubs have been deposited in the Beaty Biodiversity Museum (Marine Invertebrate Collection) at the University of British Columbia, Vancouver, Canada.

**Remarks.** This species was previously described as “*Gregarina caprellae*”, isolated from “*Caprella* sp.” collected in the Gulf of Naples [38]. The original data conformed to the gregarine we isolated from *C. alaskana*. Based on a synthesis of the molecular phylogenetic results and the morphological features of the gregarine isolated from *C. alaskana* we identified this gregarine as the previously described morphospecies “*G. caprellae*” and subsequently transfer *G. caprellae* into the genus *Heliospora*, as *H. caprellae* comb. n.

## Discussion

### Taxonomic history and recommendations

Gregarines found exclusively in the intestines of crustaceans have been classified into four main taxonomic groups (i.e., “families”): (1) the Cephaloidophoridae with about 70 species, (2) the Porosporidae with about 40 species, (3) the Uradiophoridae with about 21 species, and (4) the Ganymedidae with only two species (compare [18], [20], [24]). The validity of a fifth group of crustacean parasites is doubtful, namely the Cephalolobidae with around seven named species of gregarines (separated by Levine [39]). There are also species of gregarines known to infect crustaceans that are affiliated with different groups of gregarines that are found in a variety of different hosts (e.g. the Lecudinidae) [40]. Despite the fact that there are many gregarine morphospecies described from crustaceans, there were no confirmed molecular data available for any of them prior to this study. This paucity of molecular phylogenetic data has undoubtedly contributed to the convoluted taxonomic history of these gregarines as outlined below.

The genus *Cephaloidophora* contains the most species of any genus of gregarines that infect crustaceans, and members of this group have been characterized from a wide range of hosts, including cirripedes, decapods, and amphipods. The type species, *Cephaloidophora communis* Mawrodiadi (1908), was described from different barnacles within the genus *Balanus* and related genera such as e.g. *Megabalanus* [34]–[35], [41]; accordingly, we found *C.* cf. *communis* in two different species of barnacles: *B. glandula* from the west coast of Canada and *B. balanus* from White Sea (Arctic region of Russia). The sequences generated from *C.* cf. *communis* (from the two different hosts from different localities) were only 1.1% different; these data reinforce the morphological data that both sequences likely represent the same species rather than closely related sibling species, despite the fact these isolates came from different hosts. Although some gregarines have been experimentally observed to be host specific [42]–[44], this is does not seem to be the case for *C. communis*.

The Uradiophoridae contains two genera, namely *Heliospora* and *Uradiophora*, and five species [20]. Goodrich [36] originally established *Heliospora* for a gregarine isolated from the freshwater amphipod *Gammarus pulex*. We found *Heliospora* cf. *longissima* in two different freshwater gammarid amphipods from Lake Baikal, Siberia, Russia (*Eulimnogammarus verrucosus* and *E. vittatus*). Zvetkov [45] described a gregarine of similar appearance (*Gregarina acanthogammari*) from another freshwater amphipod (*Acanthogammarus godlevskii*) that is endemic to the same lake. This species was later placed into the genus *Heliospora* by Lipa [46]. The overall morphology of these gregarines is rather similar, but the measurements of the two gregarines we isolated conformed exactly to descriptions of the type species *H. longissima* [36], which is smaller than *H. acanthogammari*. A comparison of 1,600 SSU rDNA base pairs between the two sequences from *H.* cf. *longissima* resulted in a 0.3% sequence divergence showing that both sequences, even though from different hosts, likely represent the same species; like *C. communis*, *H. longissima* provides another example of a gregarine that seems not to be host specific.

*Heliospora longissima* has also been described from the marine amphipod *Caprella aequilibra* [47], and in this study, we were able to isolate a gregarine with very similar morphological features to *H. longissima* from *C. alaskana*; the main difference was that the overall size of the trophozoites in the gregarine we isolated was smaller than that described for *H. longissima*. Our molecular phylogenetic analysis demonstrated that the SSU rDNA sequence derived from the gregarine we isolated from *C. alaskana* clustered strongly with the two sequences of *H.* cf. *longissima*. However, a comparison of 1,606 base pairs between the SSU rDNA sequence from the gregarine isolated from *C. alaskana* and the sequences derived from *H.* cf. *longissima* (isolated from *E. verrucosus* and *E. vittatus*) resulted in 129 different bases and 16 indels (8.6% sequence divergence). These data provide compelling evidence that the gregarine isolated from *C. alaskana* is a species that is different from *H.* cf. *longissima*. In 1848, a gregarine isolated from “*Caprella* sp.” collected in the Gulf of Naples was established as “*Gregarina caprellae*” [38]. Although this species description is not very detailed, the information that was provided conformed to the gregarine we isolated from *C. alaskana*. Therefore, a synthesis of the molecular phylogenetic results and the morphological features of the gregarine isolated from *C. alaskana* led us to identify this gregarine as the previously described morphospecies “*G. caprellae*” and subsequently transfer *G. caprellae* into the genus *Heliospora*, as *H. caprellae* comb. n.

Ganymedidae currently consists of only two species, namely the type species *Ganymedes anaspidis* isolated from *Anaspides tasmaniae* collected in Tasmania, Australia [20], [48] and *G. themistos* from *Themisto libellula* collected from the Mackenzie Shelf, Canada [24]. However, six other species were previously described within *Ganymedes*: *G. apsteini* from *Calanus gracilis* and *Clausocalanus arcuicornis* in Germany and France [49], *G. eucopiae* from *Eucopia hanseni* collected in Villefranche-sur-mer, France [50]; *G. oaklandi* from *Gammarus fasciatus* collected in southern Michigan, USA [51]; *G. haeckeli* from *Sapphirina* spp. collected in Italy [49]; *G. korotneffi* from *Sergestes robustus* collected in Villefranche-sur-mer, France [50]; and *G. vibiliae* from *Vibilia armata* collected in Villefranche-sur-Mer, France [49]. Because these six species lack a ball-and-cup-like apparatus for syzygy, they were subsequently reassigned to the genus *Paraophiodina* [52]. Levine [52] also reassigned several other species, such as *Monocystis copiliae* and *Porospora pisae*, to this new genus. In the same year, Desportes et al. [53] established a new genus for the species *Porospora pisae*, namely *Thiriotia*, based on the ultrastructure of the epicytic folds and the form of lateral attachment during syzygy and placed it within the family Ganymedidae. Because of these simultaneous re-arrangements, the genus *Thiriotia* was overlooked in one of the main recent checklists of gregarine species, namely ‘An illustrated guide to the Protozoa’ [54], which follows Levine's classification scheme. The gregarine we isolated from *Pugettia gracilis* conformed to the description of *Thiriotia* by Desportes et al. [53]; this new gregarine species possessed a rare form of latero-frontal syzygy and resembled the overall morphology of the sketches of *Thiriotia* ( =  *Porospora*) *pisae* by Trégouboff [53]. This new gregarine is one of the longest gregarine species described to date, up to 2.1 mm in length, and thus differed from *Thiriotia pisae* in overall size. Accordingly, we established *Thiriotia pugettiae* n. sp. for the gregarine we isolated from *Pugettia gracilis*. We therefore, validate the genus *Thiriotia*. However, we consider the support for its replacement from the family Porosporidae too weak, taking into account the new molecular data, as *Ganymedes themistos* and *T. pugettiae* belong to different sub-clades in the phylogenetic trees (Figures 6, 7). The validation of Levine's replacements of the other six *Ganymedes* species needs further molecular phylogenetic studies on those species in question. At this point, our results confirm Grassé's gregarine classification scheme of only four subgroups (‘families’) that include gregarines that exclusively infect crustaceans.

### Molecular phylogenetic context

With only a small fraction of SSU rDNA sequences available from likely millions of species of gregarine apicomplexans (about 1,700 species partially described at the morphological level so far), this group of parasites is among the most underrepresented of all eukaryotes at the molecular level. Consequently, our understanding of gregarine diversity and taxonomy is predominantly based on morphological characteristics of trophozoites, modes of syzygy, reproductive features, and host associations (compare [23]). A comprehensive approach that combines these morphological data with molecular phylogenetic anlayses is expected to significantly improve the accuracy and utility of gregarine systematics and our understanding of parasite-host co-evolutionary relationships. For instance, phylogenetic analysis of the 82-taxon data set, including the six new gregarine sequences reported here (Figure 6); indicate that eugregarines that infect the intestines of arthropods (i.e., insects and crustaceans) do not cluster within one clade. Furthermore, all of the sequences from septate eugregarines that specifically infect the intestines of insects do not cluster within one clade either. Instead, the molecular phylogenetic data recover three major clades that include intestinal gregarines that infect arthropods: (1) terrestrial gregarine clade I consisting of neogregarines, monocystids, and a few septate and aseptate intestinal gregarines from insects; (2) terrestrial gregarine clade II consisting of other septate eugregarines from insects; and (3) a large clade of eugregarines that infect crustaceans, consisting of the species studied here plus several environmental sequences derived from aquatic habitats (Figure 6).

Although the backbone of the apicomplexan radiation is poorly resolved, these results raise the question of whether the taxonomic separation of septate and aseptate gregarines, established by Chakravarty [22], accurately reflects phylogenetic relationships. Clopton [23] recently evaluated the phylogeny of septate eugregarines using molecular data of 27 species isolated from several different insect hosts. However, this study did not analyze these data within the context of other available gregarine sequences, which prevents adequate interpretation of these data in the context of general gregarine phylogeny. Consequently, actino- and stylocephalids, as well as some related species (*Hoplorhynchus acanthatholius*, *Paraschneideria metamorphosa,* and *Prismatospora evansi*), were incorrectly inferred to form a monophyletic group with other septate eugregarines [23]. The more comprehensive molecular phylogenetic analyses presented here (Figure 6) show that septate eugregarines do not form a monophyletic group. Instead, the taxa listed above branch separately from “Terrestrial gregarine clade II” (i.e., other septate eugregarines from insects) and were scattered within “Terrestrial clade I” (also consisting of neogregarines and monocystids). This example demonstrates the importance of the taxon sample when making molecular phylogenetic inferences, even for specific groups of gregarines thought to be closely related using traditional morphological/ecological features (e.g., the presence of a septum in the trophozoites or association with insects).

### A novel SSU rDNA clade of gregarine apicomplexans from crustaceans

Seven sequences from gregarines isolated from marine and freshwater crustaceans plus eight environmental sequences formed an extremely robust clade in our molecular phylogenetic analyses (Figure 6). This “crustacean host” gregarine clade was consistently nested within a more inclusive “aquatic eugregarines” clade, albeit with weak statistical support. The “crustacean host” gregarine clade formed a weakly affiliated sister lineage to a highly supported clade consisting of marine lecudinids and urosporids isolated from a variety of different hosts, including nemerteans, polychaetes, and urochordates (Figure 6). Within marine eugregarines, there are several genera that infect specific host groups; for instance, *Lankesteria* infects urochordates [55] and *Difficilina* infects nemerteans [56]–[57]. Although some gregarine species are thought to infect only one host species [58], we have used SSU rDNA data to help demonstrate that other species can infect several different (albeit closely related) host species (e.g., *C. communis* infects several different species of *Balanus*). Accordingly, molecular phylogenetic data and DNA barcoding approaches are expected to contribute significantly to our overall understanding of host specificity in gregarines and broader patterns of host-parasite co-evolutionary history within the group. Based on the demonstrated close relationship of the gregarines within the “crustacean-host” gregarine clade we establish here the new superfamily Cephaloidophoroidea for this novel SSU rDNA clade. This is an extension of the analysis and partial systematic revision of septate gregarines performed by Clopton [23], who applied molecular data to the taxonomy of gregarines isolated from insects. According to the ICZN the name for the new superfamily is a typification of the genus that has the most representatives within the clade, namely *Cephaloidophora* Mawrodiadi, 1908.

BLAST analyses of new gregarine sequences often recover a long list of sequences derived from different environmental DNA surveys, and like gregarine sequences, most of these environmental sequences are rapidly evolving [4]. Most published environmental DNA surveys have been limited by an under-representation of available sequences from gregarine apicomplexans, especially from marine environments [1], [4], [12]–[13], [15], [59]. In Figure 7, we demonstrate that 48 environmental DNA sequences can be identified as gregarines that most likely infect crustacean hosts.

One of these environmental sequences, namely DH148-5-EKD18 is highly divergent and was sharply separated from other sequences in some of the earliest environmental DNA surveys; consequently, this sequence was interpreted as a novel, early-branching, “higher-level to new-kingdom level” taxon [1], [4], [12]–[13] that was subsequently affiliated with parabasalids [15]. Although, Cavalier-Smith [16] stated that “BOLA48 might be a gregarine, but for sure is not a new ‘higher-level' taxon”, our new data robustly demonstrate that these two particular sequences belong to a highly supported subclade of intestinal gregarines from crustacean hosts, consisting of two *Cephaloidophora* sequences from barnacles (Figures 6, 7). Most of the other environmental sequences were also discussed as ‘early branch of the eukaryotic tree’ (clone CS_R003), ‘new kingdom-level lineage BOL1’ (clones BOLA48 etc.), ‘probably novel high-level taxon united with DH148-5-EKD18’ (Sey010, Sey017), ‘independent lineage’ (DSGM-67 etc.), ‘basal-branching eukaryote lineage’ (D2P03D05, D3P05B03, D3P05G09), ‘independent lineage DH148-5-EKD18’ (Ks758762, Kn597735 etc.) [1]–[4], [10]–[14], [60]–[61].

Our data show that some of these environmental sequences represent different species of cephaloidophorid gregarines that infect various species of barnacles or other crustaceans. Our data also show that many environmental sequences (1H2dD6, 1H2dH3, etc) [62] belong to porosporids, the second strongly supported subclade within the “crustacean host” gregarine clade (superfamily Cephaloidophoroidea) that consists of the newly described species *Thiriotia pugettiae* (Figures 6, 7). Another 38 sequences (BSS 7–8 A10, BSS 0–1 44, etc) that were identified as hitherto unknown eukaryotes [63] cluster within porosporids as well (Figure 7). The third main subclade within the “crustacean host” gregarine clade (superfamily Cephaloidophoroidea) consists of two environmental sequences and three sequences from *Heliospora* (Figures 6, 7). The sequence Sey010 is most likely a partial sequence of *Heliospora longissima* based on the Kimura 2-parameter model calculations that resulted in a 0.4% sequence divergence (3 different bases out of 737). The sequence D3P05B03 differed in 18 bases out 1202 (sequence divergence 1.1%) from the sequence of *Heliospora caprellae* and could represent the same species. The data suggest that the environmental sequences within this subclade represent either the two *Heliospora* species we isolated in this study or different species of gregarines that are very closely related to species currently classified within the Uradiophoridae infecting predominantly amphipods and barnacles. Finally, only one unidentified sequence (OTU G) clusters together with *Ganymedes themistos*, forming the fourth subclade within the Cephaloidophoroidea.

### Ecology of gregarine apicomplexans from crustaceans as inferred from environmental DNA sequences

The environmental sequences belonging to the novel SSU rDNA clade were almost certainly obtained from resting stages – oocysts – within the gregarine life cycle. In general, new hosts get infected by ingestion of the oocysts. Released oocysts stay viable in sediments over a long period of time, which promotes their accumulation in these habitats. An exception is the porosporids, possessing a heteroxenous life cycle that involves crustaceans as final hosts and gastropods or bivalves as intermediate hosts. In this case, gametocysts filled with gymnocysts (i.e., zygotes either non-encysted or covered with a thin envelope) are released into the environment [64]–[65]. Edgcomb et al. [62] were able to show that DNA from resting stages, such as spores and cysts, was only present in total DNA extractions and not in cDNA extractions from the same sample because cDNA libraries reflect only metabolically active cells.

Many of the environmental sequences probably represent unknown species; however, some of them almost certainly belong to gregarine morphospecies that have been described previously. A confident species identification of such environmental sequences requires a more detailed molecular screening of already known morphospecies of gregarines from crustacean hosts. The majority of environmental sequences associated with the gregarines from crustaceans were obtained from marine, estuarine and fresh-water sediments. They were collected at sampling sites that differed geographically and ecologically: littoral zones, deep-water hydrothermal vents, cold methane seeps, Black Sea H_2_S-saturated sediments Arctic waters, Antarctic waters, subtropical waters, freshwaters in Switzerland, and Lake Baikal in Russia. Sequences related to gregarines from crustacean hosts represent about 1.5% of 10,091 rRNA phylotypes derived from a pyrosequencing study of river estuaries in Australia [66]. These data better reflect relative abundances of phylotypes in the community because 454-pyrosequencing approaches eliminate the cloning step used in other environmental DNA survey approaches [66]. These data indicate that the crustaceans living in these environments are infected by gregarines and that the gregarines are widely distributed and release oocysts that sink and accumulate in the sediment. *Heliospora longissima* sequences in Switzerland and Lake Baikal, for instance, suggest that this gregarine may infect amphipods across Eurasia including Baikal endemics. A comparison of the molecular and morphological data of *Cephaloidophora communis* indicates that this species is globally distributed. However, molecular data also indicate that that groups of closely related sequences are often found in the same location (Figure 7); however, these sequences are usually very short, so one group of sequences could represent different fragments from the same gregarine species.

There are several SSU rDNA sequences obtained from the whole gut contents of krill that are closely affiliated with gregarines: one sequence was obtained from *Euphausia superba* (Antarctic krill) [67] and four different sequences were obtained from *Meganyctiphanes norvegica* (North Atlantic krill) [68]–[69]. Both species of krill are planktonic and planktivorous; however, *E. superba* feeds on phytoplankton, while *M. norvegica* prefers to feed on copepods. The sequence AntEuk13–84 from Antarctic krill is affiliated with the subclade Cephaloidophoridae and could be a representative of the gregarine *Cephaloidophora pacifica* that infects this species frequently and sometimes very intensively [70]. The four gregarine sequences acquired from the North Atlantic krill belong to the following clades: the Cephaloidophoridae (one sequence), the Porosporidae (two sequences), and the Ganymedidae (one sequence) (Figure 7: OTUs A, E, F, and G). In addition, there are sequences from diverse lineages of phytoplankton and zooplankton, including three copepod species. Therefore, although the sequence OTU A (a close relative of AntEuk13–84) is likely derived from a gregarine that inhabits the intestines of the krill, it is also possible that some of the sequences were acquired from gregarines that inhabit the copepods that were injested by the krill. There could be at least four different gregarine species known from four different hosts: one euphausiid and three different copepods. All known gregarines from copepods were initially described as species within *Ganymedes*, but were subsequently moved, based on questionable morphological features, into *Paraophioidina* (Lecudinidae) with several species of *Porospora* [e.g., *Porospora (Thiriotia) pisae*] [52]. Because several representatives of *Paraophioidina* have the same unusual form of syzygy that is found in *Thiriotia pugettiae* and *T. pisae*, the gregarine infecting copepods could actually be porosporids or/and ganymedids. Most of the “crustacean host” gregarine sequences (superfamily Cephaloidophoroidea) have been obtained from benthic sediments and have been characterized as ‘unknown eukaryotes’. These data demonstrate that the “unkown eukaryotes” are gregarines sequences that could potentially hinder accurate estimates of crustacean diets if only a DNA-based approach is employed [67]–[69].

Some of the environmental DNA sequences within the crustacean gregarine clade were reported from hosts other than crustaceans: some gregarine sequences (Ks758762, Kn601237, Kn601238 and Kn597735) were derived from a study of deep-sea komokiaceans (foraminiferan-like amoebae) [11], two sequences were obtained from the gut contents of a bivalve (*Lucinoma aequizonata*, E4 and E35) [60], and two sequences were obtained from a marine plankton tow (DH148-5-EKD18 and BTPL20040810.0008) [4]. The gregarine sequences associated with komokiaceans most likely came from oocysts in the sediment that these organisms utilize to build their tests. The sequences from the gut contents of the bivalve could have come from transient oocysts that were caught passing through the intestine on the way out with the host feces. This is consistent with the fact that this particular bivalve (*Lucinoma aequizonata*) is a direct deposit feeder [60]. Even though it is known that porosporids use bivalve species as intermediate hosts, their developmental stages in the bivalves are found in the blood not in the intestine [64]–[65]; moreover, the sequences in question are associated with the subclade containing the Cephaloidophoridae not with the subclade of putative porosporids. The sequences found in the plankton samples could have come from planktonic crustaceans (e.g. *Ganymedes themistos* from planktonic amphipod *Themisto libellula* and *Cephaloidophora pacifica* from Antarctic krill *Euphausia superba*) [23], [67]. Some crustacean gregarine oocysts also possess projections (*Heliospora*) or filaments (*Bifilida, Pyxinioides*). These features might promote the ability to float in the water column or reduce the speed with which they sink to the bottom to increase the possibility of new planktonic host infections.

### Concluding remarks

Overall, our study shows that interpretation of environmental sequence data is greatly facilitated when representatives of poorly understood groups of eukaryotes, like different subgroups of gregarine apicomplexans, have been confidently identified and characterized at the molecular level. The highly divergent nature of gregarine sequences makes the characterization of representatives within each major subgroup especially important for this purpose. For instance, even though SSU rDNA sequences from some gregarines were known prior to this study, it was not clear whether the environmental sequences highlighted here were related to gregarines. This is because the gregarines that infect crustaceans form an extremely divergent and distinct lineage within apicomplexans (and eukaryotes as a whole) as inferred from SSU rDNA. Only after this particular lineage was characterized with SSU rDNA sequences was it possible to confidently identify the environmental sequences shown in Figures 6 and 7. Accordingly, we suspect that several more fast-evolving environmental sequences exist in GenBank that will be confidently recognized as gregarines only after species that represent major, yet unstudied, subgroups of gregarines have been collected from nature, identified, and characterized at the molecular level.

## Supporting Information

Table S1 **Complete list of closely related environmental clones forming clusters partially represented in** **Figure 7** **.** Some excessively short sequences from the 454-generated dataset from estuarine sediments in Australia are not listed.
(DOC)

Table S2 **General information about the main environmental sequences analysed.**
(DOC)
