# Supplementary material for: Identification of a Divergent Environmental DNA Sequence Clade Using the Phylogeny of Gregarine Parasites (Apicomplexa) from Crustacean Hosts
Source: PLoS One. 2011 Mar 31;6(3):e18163. doi: 10.1371/journal.pone.0018163 (PMC3069048; doi:10.1371/journal.pone.0018163)
Supplement: Table S1 — Complete list of closely related environmental clones forming clusters partially represented in Figure 7 . Some excessively short sequences from the 454-generated dataset from estuarine sediments in Australia are not listed. (DOC) [file pone.0018163.s001.doc]

**Supporting document**

Table S1. Complete list of closely related environmental clones forming clusters shown in Fig. 7. Excessively short sequences from the 454-generated dataset from estuarine sediments in Australia are partially represented here [66].

| Names of representatives shown in Fig. 7 | Name / Accession numbers of all members of the sub-cluster | Level of similarity to the first member of the cluster (BLAST data) |
| --- | --- | --- |
| BOLA048 | BOLA048 / AF372821 | 100% |
|  | BOLA374 / AF372808 | 99% |
|  | BOLA009 / AF372812 | 98% |
|  | BOLA007 / AF372823 | 98% |
|  | BOLA471 / AF372820 | 98% |
|  | BOLA012 / AF372811 | 98% |
|  | BOLA126 / AF372815 | 98% |
|  | BOLA196 / AF372824 | 98% |
|  | BOLA004 / AF372809 | 98% |
|  | BOLA604 / AF372814 | 98% |
|  | BOLA557 / AF372807 | 98% |
|  | BOLA683 / AF372822 | 98% |
|  | BOLA582 / AF372818 | 98% |
|  | BOLA546 / AF372819 | 99% |
|  | BOLA719 / AF372810 | 97% |
|  | BOLA207 / AF372813 | 97% |
|  | BOLA136 / AF372816 | 98% |
|  | BOLA543 / AF372817 | 99% |
| 1H2dH3 | 1H2dH3 / GU971820 | 100% |
|  | 1H2dC3 / GU971768 | 99% |
|  | 1H2dB5 / GU971759 | 98% |
|  | 1H2dH2 / GU971819 | 99% |
|  | 1H2dB1 / GU971752 | 99% |
|  | 1H2dG5 / GU971810 | 99% |
|  | 1H2dG3 / GU971808 | 98% |
|  | 1H2dH9 / GU971826 | 99% |
|  | 1H2dA5 / GU971747 | 99% |
|  | 1H2dE4 / GU971789 | 99% |
|  | 1H2dD3 / GU971778 | 99% |
|  | 1H2dH5 / GU971822 | 99% |
|  | 1H2dC5 / GU971770 | 99% |
|  | 1H2dB6 / GU971760 | 99% |
|  | 1H2dF7 / GU971802 | 99% |
|  | 1H2dA8 / GU971750 | 99% |
|  | 1H2dH10 / GU97181 | 99% |
|  | 1H2dF3 / GU971798 | 99% |
|  | 1H2dC6 / GU971771 | 99% |
|  | 1H2dB7 / GU971761 | 99% |
|  | 1H2dA7 / GU971749 | 99% |
|  | 1H2dE2 / GU971787 | 99% |
|  | 1H2dD2 / GU971777 | 98% |
|  | 1H2dA2 / GU971744 | 98% |
|  | 1H2dC4 / GU971769 | 98% |
|  | 1H2dC2 / GU971767 | 98% |
|  | 1H2dG6 / GU971811 | 98% |
|  | 1H2dG8 / GU971813 | 98% |
|  | 1H2dE5 / GU971790 | 98% |
|  | 1H2dE3 / GU971788 | 98% |
|  | 1H2dA1 / GU971741 | 98% |
|  | 1H2dC1 / GU971764 | 98% |
|  | 1H2dB9 / GU971763 | 99% |
|  | 1H2dB3 / GU971757 | 99% |
|  | 1H2dD5 / GU971780 | 99% |
|  | 1H2dD9 / GU971784 | 98% |
|  | 1H2dE11 / GU971786 | 98% |
|  | 1H2dD7 / GU971782 | 98% |
|  | 1H2dB8 / GU971762 | 98% |
|  | 1H2dB9_1 / GU971830 | 99% |
|  | 1H2dA12_1 / GU971829 | 99% |
|  | 1H2dA10_1 / GU971827 | 99% |
|  | 1H2dH1 / GU971815 | 99% |
|  | 1H2dA11_1 / GU971828 | 99% |
|  | 1H2dF12 / GU971796 | 97% |
|  | 1H2dF5 / GU971800 | 99% |
|  | 1H2dE6 / GU971791 | 99% |
|  | 1H2dE7 / GU971792 | 98% |
|  | 1H2dH6 / GU971823 | 98% |
|  | 1H2dF8 / GU971803 | 98% |
|  | 1H2dD6 / GU971781 | 97% |
| PS13C11 | PS13C11 / GU072510 | 100% |
|  | PS13H7 / GU072531 | 100% |
|  | PS13F11 / GU072520 | 100% |
|  | PS13H8 / GU072532 | 99% |
|  | PS13D8 / GU072515 | 99% |
| GPS3H12 | GPS3H12 / GU072292 | 100% |
|  | GPS3H1 / GU072284 | 99% |
| G513C3 | G513C3 / GU072099 | 100% |
|  | G513E11 / GU072108 | 99% |
| Kn601238 | Kn601238 / FJ646765 | 100% |
|  | Kn601231 / FJ646766 | 99% |
|  | Kn601239 / FJ646767 | 99% |
|  | Kn601232 / FJ646764 | 99% |
| Kn597735 | Kn597735 / FJ646770 | 100% |
|  | Kn597731 / FJ646769 | 99% |
|  | Kn597625 / FJ646771 | 99% |
|  | Kn597621 / FJ646768 | 98% |
| BSS 7-8 A10 | BSS 7-8 A10 / GU474155 | 100% |
|  | BSS 7-8 C11 / GU474161 | 100% |
|  | BSS 7-8 C12 / GU474158 | 100% |
|  | BSS 7-8 D03 / GU474156 | 100% |
|  | BSS 7-8 E02 / GU474164 | 100% |
|  | BSS 7-8 E06 / GU474153 | 100% |
|  | BSS 7-8 F02 / GU474162 | 100% |
|  | BSS 7-8 F03 / GU474163 | 100% |
|  | BSS 7-8 F08 / GU474157 | 100% |
|  | BSS 7-8 F11 / GU474165 | 100% |
|  | BSS 7-8 G03 / GU474154 | 100% |
|  | BSS 7-8 H09 / GU474160 | 100% |
|  | BSS 7-8 H11 / GU474159 | 100% |
|  | BSS 0-1 11 / GU474110 | 100% |
|  | BSS 0-1 67 / GU474111 | 99% |
|  | BSS 0-1 103 / GU474112 | 99% |
|  | BSS 0-1 143 / GU474109 | 99% |
|  | BSS 0-1 10 / GU474100 | 97% |
|  | BSS 0-1 23 / GU474101 | 97% |
|  | BSS 0-1 36 / GU474102 | 97% |
|  | BSS 0-1 47 / GU474104 | 97% |
|  | BSS 0-1 78 / GU474106 | 97% |
|  | BSS 0-1 79 / GU474103 | 97% |
|  | BSS 0-1 102 / GU474105 | 97% |
|  | BSS 0-1 123 / GU474107 | 97% |
|  | BSS 0-1 127 / GU474108 | 97% |
| BSS 0-1 44 | BSS 0-1 44 / GU474055 | 100% |
|  | BSS 0-1 97 / GU474056 | 100% |
|  | BSS 7-8 F07 / GU474148 | 100% |
|  | BSS 7-8 G09 / GU474149 | 100% |
|  | BSS 7-8 A08 / GU474200 | 99% |
|  | BSS 7-8 B01 / GU474147 | 99% |
|  | BSS 7-8 E07 / GU474150 | 99% |
|  | BSS 7-8 G07 / GU474151 | 99% |
|  | BSS 7-8 H01 / GU474146 | 99% |
|  | BSS 7-8 H04 / GU474199 | 99% |
|  | BSS 7-8 H05 / GU474145 | 99% |
|  | BSS 7-8 G12 /GU474144 | 98% |
| E88AHAQ01AFY6Z | E88AHAQ01AFY6Z / FJ920577 | 100% |
|  | E88AHAQ02GB4Q2 / FJ992622 | 100% |
|  | E88AHAQ02IWJRC / FJ996071 | 99% |
|  | E88AHAQ02IDPEF / FJ995317 | 99% |
|  | E88AHAQ02HVF43 / FJ994620 | 99% |
|  | E88AHAQ02HKE1N / FJ994222 | 99% |
|  | E88AHAQ02H3ZPB / FJ993675 | 99% |
|  | E88AHAQ02GLS6A / FJ993007 | 99% |
|  | E88AHAQ02GH8XD / FJ992833 | 99% |
|  | E88AHAQ02GD6T1 / FJ992698 | 99% |
|  | E88AHAQ02GBSZG / FJ992651 | 99% |
|  | E88AHAQ02G2J21 / FJ992345 | 99% |
|  | E88AHAQ02FYM5H / FJ992235 | 99% |
|  | E88AHAQ02FS003 / FJ992086 | 99% |
|  | E88AHAQ01B09W6 / FJ987357 | 99% |
|  | E88AHAQ02JYNUX / FJ930038 | 99% |
|  | E88AHAQ02JGWJ7 / FJ929727 | 99% |
|  | E88AHAQ02I1JKB / FJ928800 | 99% |
|  | E88AHAQ02GR75O / FJ927873 | 99% |
|  | E88AHAQ02G9038 / FJ927443 | 99% |
|  | E88AHAQ02FZR0E / FJ927215 | 99% |
|  | E88AHAQ02FJFLI / FJ926839 | 99% |
|  | E88AHAQ02FIG5F / FJ926820 | 99% |
|  | E88AHAQ02F3TJ5 / FJ926548 | 99% |
|  | E88AHAQ01BS685 / FJ922703 | 99% |
|  | E88AHAQ02JG3PQ / FJ996489 | 99% |
|  | E88AHAQ02I8MTD / FJ995124 | 99% |
|  | E88AHAQ02IGVIH / FJ995439 | 98% |
|  | E88AHAQ02JV6UK / FJ997071 | 96% |
| E88AHAQ01ACY6H | E88AHAQ01ACY6H / FJ920469 | 100% |
|  | E88AHAQ02JA4OS / FJ996255 | 99% |
|  | E88AHAQ02GJKPF / FJ992922 | 99% |
|  | E88AHAQ01DUIHC / FJ990878 | 99% |
|  | E88AHAQ01DBL8Q / FJ990202 | 99% |
|  | E88AHAQ01C3Q09 / FJ988684 | 99% |
|  | E88AHAQ01BB0PQ / FJ987689 | 99% |
|  | E88AHAQ01BAWV7 / FJ987688 | 99% |
|  | E88AHAQ01AUOYG / FJ987195 | 99% |
|  | E88AHAQ01DRNO9 / FJ925464 | 99% |
|  | E88AHAQ01AS7YH / FJ921108 | 99% |
|  | E88AHAQ01CX7CB / FJ989735 | 99% |
|  | E88AHAQ01C2O3L / FJ988638 | 99% |
|  | E88AHAQ01BWJKH / FJ988431 | 99% |
|  | E88AHAQ01BTW2H / FJ922780 | 99% |
|  | E88AHAQ01EB1E7 / FJ991068 | 99% |
|  | E88AHAQ01DVBAE / FJ990913 | 99% |
|  | E88AHAQ01CYWWZ / FJ924435 | 99% |
|  | E88AHAQ01EJLGM / FJ991323 | 99% |
|  | E88AHAQ01A8GWR / FJ986732 | 97% |
| E88AHAQ01BQ67F | E88AHAQ01BQ67F / FJ922621 | 100% |
|  | E88AHAQ01AEZWF / FJ986809 | 100% |
|  | E88AHAQ01EQ0UT / FJ991490 | 99% |
|  | E88AHAQ01DVEJA / FJ990917 | 99% |
|  | E88AHAQ01C51GT / FJ988735 | 99% |
|  | E88AHAQ01BTIUD / FJ988335 | 99% |
|  | E88AHAQ01B4IIP / FJ987478 | 99% |
|  | E88AHAQ01AHGXQ / FJ986858 | 99% |
|  | E88AHAQ01BSG8K / FJ922716 | 99% |
|  | E88AHAQ01EJ7OU / FJ991315 | 99% |
|  | E88AHAQ01CWLRZ / FJ989711 | 99% |
|  | E88AHAQ01CDJ64 / FJ988998 | 99% |
|  | E88AHAQ01DXEAU / FJ990977 | 98% |
| E88AHAQ01B185I | E88AHAQ01B185I / FJ921537 | 100% |
|  | E88AHAQ01EFWOL / FJ991223 | 99% |
|  | E88AHAQ01A9X6S / FJ920445 | 99% |
|  | E88AHAQ01ANTT3 / FJ920909 | 97% |
|  | E88AHAQ01A6GBF / FJ986687 | 97% |
|  | E88AHAQ01D3APW / FJ924581 | 97% |
|  | E88AHAQ01ACRTT / FJ920463 | 97% |
|  | E88AHAQ01D37H3 / FJ989915 | 96% |
|  | E88AHAQ01DIL3J / FJ990456 | 96% |
| E88AHAQ01AX26E | E88AHAQ01AX26E / FJ921343 | 100% |
|  | E88AHAQ02JCD7L / FJ996351 | 99% |
|  | E88AHAQ02I5209 / FJ994985 | 99% |
|  | E88AHAQ02HGQ3Q / FJ994110 | 99% |
|  | E88AHAQ01ESDBK / FJ926372 | 99% |
|  | E88AHAQ02JCPJ7 / FJ996364 | 98% |
|  | E88AHAQ02I4V08 / FJ994977 | 98% |
| E88AHAQ01B44VL | E88AHAQ01B44VL / FJ987469 | 100% |
|  | E88AHAQ01DGQWN / FJ990395 | 100% |
|  | E88AHAQ01BWXQN / FJ988443 | 99% |
|  | E88AHAQ01BGGQS / FJ922196 | 99% |
|  | E88AHAQ01C2QKN / FJ988641 | 98% |
| E88AHAQ01CCUKD | E88AHAQ01CCUKD / FJ923572 | 100% |
|  | E88AHAQ01BMAHQ / FJ988083 | 99% |
|  | E88AHAQ01EMQOI / FJ991404 | 98% |
|  | E88AHAQ01B1TEK / FJ987399 | 98% |
| E88AHAQ02GMEZM | E88AHAQ02GMEZM / FJ993023 | 100% |
|  | E88AHAQ01A9F02 / FJ920429 | 99% |
