# Supplementary material for: Identification of a Divergent Environmental DNA Sequence Clade Using the Phylogeny of Gregarine Parasites (Apicomplexa) from Crustacean Hosts
Source: PLoS One. 2011 Mar 31;6(3):e18163. doi: 10.1371/journal.pone.0018163 (PMC3069048; doi:10.1371/journal.pone.0018163)
Supplement: Table S2 — General information about the main environmental sequences analysed. (DOC) [file pone.0018163.s002.doc]

Table S2. General information about the main environmental sequences analysed.

| Name | Sample | Reference | Author’s characteristic of sequence |
| --- | --- | --- | --- |
| DH148-5-EKD18 | marine plankton, deep sea, Antarctic polar front | López-García et al. (2001) | new kingdom-level lineage |
| CS_R003 | marine, deep sea sediment, Guaymas Basin hydrothermal vent, Gulf of California, Pacific, subtropical | Edgcomb et al. (2002) | early branch of the eukaryotic tree |
| BOLA48, etc.  (total: 18 sequences, see Fig. 7 and Table S1) | marine, low tide sediment, Bolinas Tidal Flat, Bolinas, California, Pacific, subtropical | Dawson,& Pace (2002) | new kingdom-level lineage BOL1 |
| Sey010,  Sey017 | Fresh water, river sediment, Europe, Switzerland | Berney et al. (2004) | probably novel high-level taxon |
| E4,  E35 | marine, gut contents of bivalve mollusc *Lucinoma aequizonata*, Santa Barbara Basin, California, Pacific, subtropical | Duplessis et al. (2004). | characterized by BLAST hits only |
| DSGM-67 – 73, CYSGM-20, DM2-SGM41 | marine, oxygen-depleted sediment (microbial mats) from a deep-sea methane cold seep of Sagami Bay, Japan, East Pacific | Takishita et al. (2007) | independent lineage |
| D2P03D05, D3P05B03, D3P05G09 | oxygen-depleted intertidal marine sediment, Greenland, Arctic | Stoeck et al.(2007) | basal-branching lineage |
| Ks758762, Kn601237, Kn601238, Kn597735, etc.  (total: 10 sequences, see Fig. 7 and Table S1 | marine, Weddell Sea, deep-sea soil associated with Komokiacea, Antarctic | Lecroq et al. (2009) | independent lineage DH148-5-EKD18 |
| G513C3,  GPS3B12, GPS3H12,  PS13F11, etc.  (total: 8 sequences, see Fig. 7 and Table S1) | intertidal marine sediment, Arabian Sea, Indian Ocean, subtropical | Jebaraj et al. (2010) | characterized by BLAST hits only |
| BTPL20040810.0008 | subsurface seawater, estuary, temperate Atlantic | Caron et al. (UNPUBLISHED: “*in prep*.”) | **?** |
| 1H2dD6,  1H2dH3, etc.  (total: 51 sequences, see Fig. 7 and Table S1) | marine sediment, Pacific | Edgcomb et al. (2010) | Alveolata (spores of apicomplexans) |
| BSS 0-1 etc,  BSS 7-8 etc.  (total: 38 sequences, see Fig. 7 and Table S1) | anoxic deep-sea sediments: top 1 cm or 7-8 cm layers, Black-Sea | Corinaldesi et al. (2010) | hitherto unknown eukaryotic kingdoms |
| AntEuk13-84 | gut contents of Southern krill *Euphausia superba*, Antarctic plankton | Martin et al. (2006) | unidentified eukaryote |
| OTUs A, E, F, G | gut contents of Northern krill *Meganyctiphanes norvegica*, North Atlantic plankton | Cleary et al. (2010, 2011) [abstracts of presentations] | presumably: ‘unknown benthic microeukaryotes’ (GenBank reference: uncultured eukaryote clone) |
| E88AHAQ01AFY6Z,  E88AHAQ02HKIFZ, etc.  (total: 91 pyrosequences, see Fig. 7 and Table S1) | estuarine sediment, Australia | Chariton et al. (2010) | Not specified  (GenBank reference: uncultured eukaryote clone) |
